# Projecting RNA measurements onto single cell atlases to extract cell type-specific expression profiles using scProjection

**DOI:** 10.1038/s41467-023-40744-6

**Published:** 2023-08-25

**Authors:** Nelson Johansen, Hongru Hu, Gerald Quon

**Affiliations:** 1 Graduate Group in Computer Science, University of California, Davis, Davis, CA USA; 2 Integrative Genetics and Genomics Graduate Group, University of California, Davis, Davis, CA USA; 3 Department of Molecular and Cellular Biology, University of California, Davis, Davis, CA USA

**Keywords:** Machine learning, Data integration, Gene expression, Computational science, RNA sequencing

## Abstract

Multi-modal single cell RNA assays capture RNA content as well as other data modalities, such as spatial cell position or the electrophysiological properties of cells. Compared to dedicated scRNA-seq assays however, they may unintentionally capture RNA from multiple adjacent cells, exhibit lower RNA sequencing depth compared to scRNA-seq, or lack genome-wide RNA measurements. We present scProjection, a method for mapping individual multi-modal RNA measurements to deeply sequenced scRNA-seq atlases to extract cell type-specific, single cell gene expression profiles. We demonstrate several use cases of scProjection, including identifying spatial motifs from spatial transcriptome assays, distinguishing RNA contributions from neighboring cells in both spatial and multi-modal single cell assays, and imputing expression measurements of un-measured genes from gene markers. scProjection therefore combines the advantages of both multi-modal and scRNA-seq assays to yield precise multi-modal measurements of single cells.

## Abstract (web summary)

Many expression deconvolution approaches have been developed to estimate % RNA contributions of diverse cell types to mixed RNA measurements. Here, the authors have developed a complementary approach called scProjection to recover cell type-specific expression profiles from mixed RNA measurements.

## Introduction

In recent years, there has been a surge in the number and size of atlasing efforts across tissues, conditions, and species^1–4^, driven by the high throughput nature of single cell- and nucleus-RNA sequencing (sc/snRNA-seq) technologies. These technologies are now routinely used to generate atlases on the scale of up to millions of cells^3,5–7^, in order to maximize the discovery of novel cell types and characterize the transcriptional heterogeneity of individual cell types within samples. One of the limitations of the sc/snRNA-seq technologies, however, is that they only measure RNA content. To address this limitation, there are a growing number of single-cell *resolution* assays that simultaneously measure RNA content as well as other cellular annotations and modalities. For example, spatial transcriptomic sequencing assays such as Slide-seq^8^ and LCM-seq^9^ record both the spatial position and RNA measurements of individual spots on a sample. There are also multi-modal assays such as Patch-seq^10,11^ that measure cellular phenotypes in addition to local RNA content, enabling the identification of connections between molecular and cellular phenotypes of neurons.

However, single-cell resolution assays often trade precision in their RNA measurements in exchange for collecting additional data modalities; consider the following three examples. First, for spatial transcriptome sequencing assays such as LCM-seq or Slide-seq, RNA is extracted from spots of pre-defined size and location on tissue in order to infer spatial gene expression patterns. Because the spots are not lined up with the locations of individual cells, individual spots often capture RNA from multiple adjacent cells by chance, leading to imprecise spatial expression patterns. Second, for the Patch-seq assay that jointly measures RNA and electrophysiological properties of neurons, the collected RNA can include contributions from the target neuron as well as surrounding cells when performed on in vivo or ex vivo slices^12^. This contamination of the measured RNA leads to lower power to identify relationships between gene expression levels and electrophysiological features of neurons. Third, for spatial transcriptome technologies such as MERFISH^13^, in practice, only a few hundred selected genes in the genome can be measured in a tissue. This prevents the detection of spatial expression patterns of genes not directly selected for measurement.

Here we present scProjection, a unified framework for addressing imprecise single-cell resolution assays. In summary, scProjection uses deeply sequenced single cell atlases to improve the precision of individual single-cell resolution RNA measurements. It does so by jointly performing two tasks: deconvolution (estimating % RNA contributions of each of a set of cell types to a single RNA measurement) and projection (extracting a cell type-specific gene expression profile for each of a set of cell types from an RNA measurement). We show that scProjection achieves state-of-the-art performance in both the deconvolution and projection tasks. Then, we illustrate the use of scProjection for improving analyses of data generated from the three examples of imprecise single-cell resolution assays described above. First, we show scProjection analysis of spatial transcriptomes increases the detection of cell type-specific spatial gene expression patterns across diverse tissues in the brain and intestinal villus. Second, we show scProjection can impute the expression levels of genes not directly measured via spatial transcriptome technologies such as MERFISH^13^. Finally, we show scProjection can separate RNA contributions of the target neuron from neighboring glial cells when analyzing Patch-seq data, leading to more accurate prediction of one data modality (electrophysiological response) from another (RNA expression levels). We conclude that integrating deep single-cell atlases with single and multimodal cell resolution assays can therefore combine the advantages of both sequencing approaches to study single cells.

## Results

### scProjection overview

**Fig. 1:**
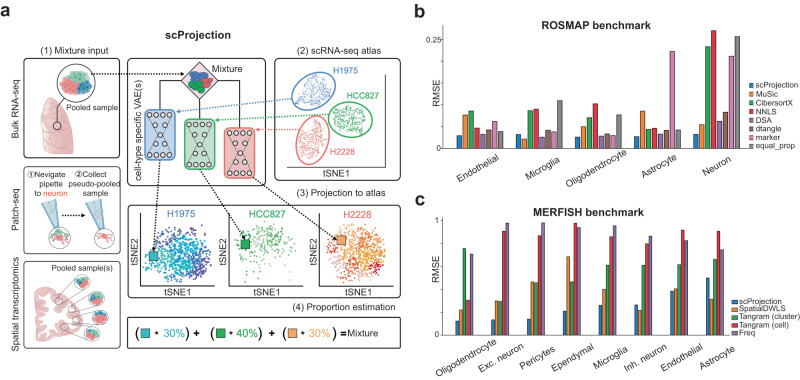
RNA projection and cell type abundance estimation with scProjection. **a** (1) The primary input to scProjection consists of one or more RNA measurements originating from mixtures of cells assayed using bulk RNA-seq, multi-modal assays or spatial transcriptomics. (2) The secondary input to scProjection is a single-cell atlas from the same region or tissue as the mixture samples and contains the same cell types present in the mixture samples. For each of the annotated cell types in the single-cell atlas, a variational autoencoder is trained to capture within-cell type variation in expression. (3, 4) scProjection uses the variational autoencoder to extract cell type-specific contributions to each mixed sample, as well as the % RNA contribution of each of those cell types to the mixture. **b** Bar plots indicate the root-mean-square error (RMSE) in predicted cell type abundances for each deconvolution method on the ROSMAP (Patrick et al. 2020) benchmark data; grey bars represent the error of a baseline approach (equal_prop) of predicting equal RNA contributions from each cell type. **c** Bar plots indicate the RMSE in the estimated cell type proportion for each deconvolution method on the spatial transcriptome-based benchmarking data (Moffitt et al. 2018). Purple bars (Freq) represent the error of a baseline approach of predicting proportion based on the frequency of each cell type in the MERFISH RNA measurements, based on the original authors’ labels.

The scProjection model and workflow is illustrated in Fig. 1a. scProjection assumes that one or more mixed RNA samples ${{{{{{\bf{b}}}}}}}_{n}$ from an imprecise single cell resolution assay are available as input, as well as a deeply sequenced single-cell atlas that profiles the same cell types as those contributing to the mixed RNA samples. Typical single-cell resolution assays of interest include spatial transcriptome assays such as LCM-seq, Slide-seq or MERFISH, multimodal assays such as Patch-seq, or classical bulk RNA-seq. As output, scProjection simultaneously projects each RNA sample ${{{{{{\bf{b}}}}}}}_{n}$ onto each component cell population $k$ within the single cell atlas to estimate the average cell state (expression profile) of that cell type in the mixed sample $({{{{{{\bf{x}}}}}}}_{n , k})$, as well as to estimate the relative abundance of that cell type $({\alpha }_{i,k})$. scProjection, therefore, balances selecting sets of cell states ${{{{{{\bf{x}}}}}}}_{n,k}$ that help minimize reconstruction error of the input mixed RNA measurement ${{{{{{\bf{b}}}}}}}_{n}$, with selecting cell states that are frequently occurring in the single cell atlas (e.g. the prior).

scProjection uses individual variational autoencoders^14^ (VAEs) trained on each cell population within the single cell atlas to model within-cell type expression variation and delineate the landscape of valid cell states^15^, as well as their relative occurrence. Here, a valid cell state for a cell type $k$ is defined as a genome-wide gene expression profile that has either been directly measured in the single cell atlas or is inferred to be feasible based on gene co-expression patterns observed in measured cells. In practice, we ignore projections ${{{{{{\bf{x}}}}}}}_{n,k}$ when the corresponding predicted cell type abundances ${\alpha }_{i,k}$ is small (e.g. <5%).

With respect to the deconvolution task, scProjection outperforms both dedicated deconvolution approaches (CIBERSORTx^16^, MuSic^17^, dtangle^18^, DSA^19^, NNLS^20^) as well as the deconvolution approaches implemented by spatial transcriptome methods (SpatialDWLS^21,22^ and Tangram^23^) across multiple datasets and benchmarks, such as CellBench^24^, ROSMAP^25^, and MERFISH^26^ (Fig. 1b, c, Figs. S1–S2). We also performed experiments in which we removed a contributing cell type from the single cell atlas, and found that scProjection assigns the missing cell type’s contributions to the most closely related cell type (Fig. S3), instead of equally distributing that weight to all represented cell types. Our results therefore suggest scProjection is state-of-the-art with respect to identifying total RNA contributions from different cell types to RNA mixtures. In the remainder of this paper, we focus on the task of projection (finding the average cell state ${{{{{{\bf{x}}}}}}}_{n,k}$ of each contributing cell type $k$ to a mixed sample ${{{{{{\bf{b}}}}}}}_{n}$) because it has received less attention than the problem of deconvolution (estimating the relative abundance ${\alpha }_{i,k}$ of cell type $k$ in mixed sample ${{{{{{\bf{b}}}}}}}_{n}$).

### Projections distinguish within cell type variation in gene expression patterns

**Fig. 2:**
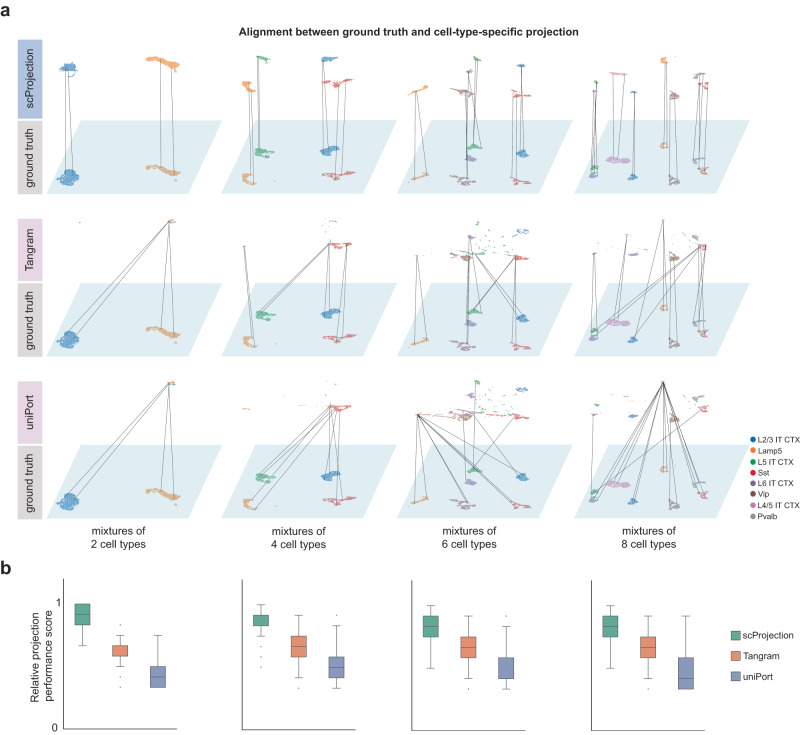
scProjection recovers cell type-specific RNA contributions to synthetic mixed RNA samples through projection. **a** Joint UMAP visualization of the individual cells contributing to the 5000 synthetic RNA mixtures (ground truth, bottom rows), as well as the projection of each of the 5000 synthetic RNA mixtures to each contributing cell type from a scRNA-seq atlas (top rows). Each column represents the data for RNA mixtures with different numbers of contributing cell types, while each pair of rows represents a prediction method. Note that for the ground truth plots on the bottom rows, there are 5000 x (# of contributing cell types) points in the visualization; the number of points is the same for the prediction plots on the top rows. Note there are overlapping points because the single cell atlases used to generate simulated mixtures often contain fewer than 5000 cells per cell type. Cells are colored by cell type. Line segments represent pairs of cells for the same simulated RNA mixture, where one cell is the ground truth cell that contributed to an RNA mixture, and the other cell is the projection of the RNA mixture to the cell type of the ground truth cell. Vertical line segments represent more accurate predictions. **b** Box plots indicating the average (across *n* = 5000 cells per cell type) cell-wise relative projection performance scores of scProjection (green), Tangram (orange), and uniPort (blue) for each mixture type based on the metrics CC, FOSCTTM, RMSE, and JSD shown in Fig. S4 (see Methods). In the box plots, the minima, maxima, centerline, bounds of box, and whiskers represents minimum value in the data, maximum value in the data, median, upper and lower quartiles, and 1.5x interquartile range, respectively.

To establish the accuracy of scProjection for mapping RNA samples to the correct transcriptional state for each contributing cell type, we simulated mixed RNA samples by combining transcriptional states of multiple cell types. We generated four sets of 5000 RNA mixtures each, where each set corresponds to mixing a single cell from each of either 2, 4, 6 or 8 mouse primary motor cortex neuronal cell types^5^. Figure 2a visualizes the predicted and ground truth cell states for each of the 5000 simulated mixtures for each of the four mixture sets, and demonstrates how the set of projected transcriptional states of scProjection more closely resemble the pool of transcriptional states used to generate the simulated mixtures, compared to Tangram and uniPort. We defined a “relative projection performance score” (Fig. 2b) by combining each method’s ranking in terms of four individual similarity (or distance) metrics (CC, RMSE, JSD, and FOSCTTM) (Fig. S4), analogous to an established composite metric for measuring the performance of transcript distribution prediction and deconvolution^27^ (see Methods). When comparing individual projections of RNA mixtures to the ground truth cell states used to generate the mixtures, scProjection overall projection performance score was on average 27% and 79% higher than Tangram and uniPort, respectively (Fig. 2b). In particular, when looking at the individual performance metrics that contribute to overall projection performance score, we observed the correlation between projected and ground truth for scProjection was higher on average by 38% and 42% compared to Tangram and Uniport, respectively (Fig. S4). Strikingly, scProjection performance was better for individual mixture sets as well (Fig. 2b), suggesting that even when individual cell types contribute less RNA to the total mixture (8 cell type mixtures), scProjection is still able to recover the original transcriptional state more accurately. These results indicate that scProjection can better resolve cell state when projecting complex mixed RNA samples to their component cell types compared to current state-of-the-art methods.

### Imputing spatial gene expression patterns using fluorescence-based spatial transcriptomics

Having shown that scProjection can identify transcriptional states of individual cell types from a mixed RNA measurement, we next explored our first application: inference of cell type-specific expression patterns in fluorescence in situ hybridization (FISH)-based spatial transcriptome measurements. FISH-based technologies such as MERFISH^28^ and osmFISH^29^ have recently been used to profile tens to hundreds of genes’ expression levels in the mouse brain^26,30^ and other tissues and species^31–33^.The relatively small profiled gene panels in these studies is due to necessary trade-offs between capture efficiency and the number of genes profiled^34^, and lead to a limited identification of genome-wide spatial expression patterns. The scProjection framework can be modified to project RNA measurements based on small gene panels onto a single cell atlas to infer a genome-wide gene expression profile, which in essence imputes the un-measured genes. We therefore reasoned we could use scProjection to infer genome-wide expression profiles from MERFISH/osmFISH data, in order to then identify spatial expression patterns across all genes.

**Fig. 3:**
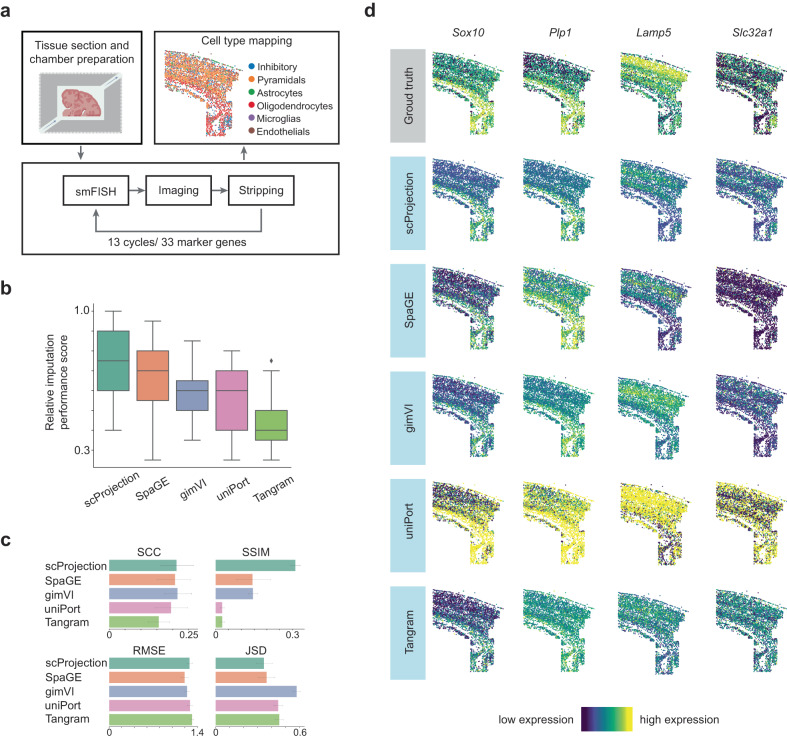
scProjection accurately imputes the distribution of unseen RNA transcripts based on small gene panels. **a** Overview of the Codeluppi et al.^29^ osmFISH experiment on mouse cortical brain slices (preparation cartoon was created with BioRender.com). **b** Box plot of relative imputation performance scores of methods for imputing spatial gene-expression patterns for *n* = 33 genes in a leave-one-out framework. In the box plots, the minima, maxima, centerline, bounds of box, and whiskers represents minimum value in the data, maximum value in the data, median, upper and lower quartiles, and 1.5x interquartile range, respectively. **c** Bar plots showing the average Spearman’s correlation coefficient (SCC), structural similarity index measure (SSIM), root-mean-square error (RMSE), and Jensen–Shannon distance (JSD) component scores across held-out genes (*n* = 33) for each method for the experiment performed in **b**, and the error bars indicates the standard deviation. Higher scores in SCC and SSIM (top row) and lower scores in RMSE and JSD (bottom row) indicate better imputation performance. **d** Actual and predicted spatial expression patterns of selected genes.

We first evaluated the accuracy of scProjection at imputing unmeasured gene expression from spatial transcriptomics data. We obtained data from a recent osmFISH study of the mouse primary motor cortex (MOp) by Codeluppi et al. ^29^ (Fig. 3a) and an unpaired mouse cortical scRNA-seq atlas^35^ to form the input to scProjection. We evaluated imputation accuracy by repeatedly holding out a single measured gene from the original 33-gene panel, and using the other 32 genes to impute the expression of the held-out gene. We compared scProjection against state-of-the-art spatial imputation methods established in a recent comprehensive benchmark study^27^, including Tangram^23^, gimVI^36^, SpaGE^37^, and uniPort^38^ using an established spatial transcriptome composite relative imputation performance score consisting of the ranking of four different measures of similarity and distance (SCC, SSIM, RMSE, JSD; see Methods)^27^. Based on the composite score, scProjection outperformed all other imputation methods by an average of 38% (Fig. 3b). Looking at the individual component scores, scProjection performs significantly better than other approaches in the structural similarity index measure (SSIM), a measure traditionally used to judge pairwise image similarity (Fig. 3c). Figure 3d presents examples of the measured and predicted spatial expression patterns of *Sox10*, *Plp1*, *Lamp5,* and *Slc32a1*, and illustrates how scProjection broadly captures the per-gene spatial expression patterns present in the ground truth more closely compared to other methods.

**Fig. 4:**
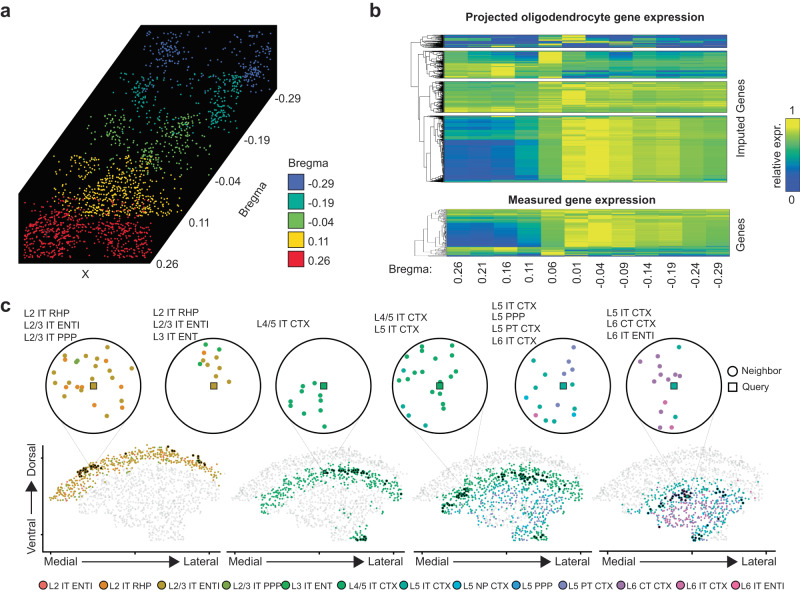
Transcriptome inference and high-resolution label transfer identifies spatial expression patterns in the brain. **a** Stacked MERFISH images of oligodendrocyte populations identified according to dominant cell type predicted by scProjection across anterior to posterior slices from Moffitt et al. Images are ordered by Bregma value. **b** Heatmap visualizing the gene-specific spatial expression patterns within the oligodendrocytes of imputed (top) and measured (bottom) genes. **c** MERFISH image of a single slice of the mouse cortex from Zhang et al., replicated four times to highlight neurons from different layers. Six examples of statistically significant spatial motifs (recurring neighborhoods) are illustrated in the circle plots. Spatial motifs were identified by first categorizing each individual cell (‘query’, squares) in the MERFISH dataset into a neighborhood type based on the number and cell types of neighboring cells (‘neighbor’, circles) within a radius around the query, then using permutation tests to determine whether there are specific neighborhood types that occur more frequently than expected by chance.

Having demonstrated scProjection can impute gene expression levels accurately, we applied scProjection to MERFISH data collected by Moffitt et al.^26^ on neurons from the hypothalamic preoptic region of the mouse brain, in order to identify novel spatial expression patterns. In this study, 155 marker genes were measured across millions of neurons, while a matched scRNA-seq cell atlas was also generated. We first applied scProjection to perform deconvolution in order to identify the major cell type contributing to each MERFISH measurement. Labeling each MERFISH measurement by the cell type predicted to contribute the most RNA, we found scProjection recovered the spatial organization of oligodendrocytes across slices from the mouse brain defined by Bregma indices (Fig. 4a). Bregma defines the distance to the anatomical position at which the coronal suture is intersected perpendicularly by the sagittal suture. We observe that the oligodendrocytes spatially organize into one cluster at Brega 0.26, then diverges into two populations by Bregma −0.29.

To explore potential functional implications of the segmentation of oligodendrocytes from one into two spatial regions, we used scProjection to project each MERFISH measurement to the oligodendrocyte population in the scRNA-seq atlas, and extracted Bregma index-specific expression patterns of oligodendrocytes. We identified imputed gene expression with clear differential expression patterns across the two distal Bregma indices (Fig. 4b). Of particular note are *Calca* and *Dpp10*, both of which are associated with oligodendrocyte differentiation that occurs along the Bregma axis, with immature and mature oligodendrocytes occupying separate compartments of the hypothalamus^26^. Neither of these markers belonged to the 155 marker gene set measured by MERFISH in the original study. Our results therefore show that scProjection can help identify genes with spatially distinct expression patterns even for genes not measured in the original assay.

### Identification of global spatial expression patterns and cell type motifs in the brain

We next wondered whether projection could facilitate the identification of spatial patterns of cell type organization in the brain, as opposed to spatial patterns of single gene expression as we explored in the previous section. The identification of spatial patterns is a task often performed at the individual gene level, and many approaches have been developed to identify non-random spatial single-gene expression patterns^39,40^. Spatial patterning within tissues extends beyond the level of individual gene expression patterns, however. At a coarse level, the mammalian brain organizes neurons into functional neighborhoods that vary with cortical depth^13,30^. Interneurons from different layers of the cortex are widely recognized as distinct in their transcriptome and function^1,5,41^. We hypothesized that there might be more localized organizational features in the brain, involving potentially small groups of cell types that frequently spatially co-occur together. We term these groups of co-occurring cells “spatial motifs”.

To test this hypothesis of the existence of spatial motifs, we set out to identify spatial motifs in the brain by analyzing MERFISH data from a recent study by Zhang et al. ^30^, in conjunction with a million-neuron atlas of the mouse primary motor cortex (MOp) from Yao et al. ^5^. We used scProjection to infer a revised high-resolution cell type label for each MERFISH measurement by projecting MERFISH measurements to the snRNA-seq atlas and assigning discrete labels based on the taxonomy of Yao et al., which defines 129 cell types under the broader categories of glutamatergic, GABAergic, and non-neuronal subtypes. We defined spatial motifs as spatial neighborhoods consisting of a specific set of cell types that are unlikely to co-occur by chance. We assigned each cell into a spatial neighborhood type based on the observed cell types within a 100um distance. We then counted the number of cells assigned to each spatial neighborhood type, and permuted the cell type labels 1,000,000 times, making sure cell labels only permute across cells of the same layer. Through comparison with simulated neighborhood occurrences, we identified a diverse set of 19 significant neighborhood types (permuted *p* < 5 × 10^−8^, at least 50 cells assigned to that neighborhood type) ranging from homogenous L4/5 populations to neighborhoods that exist on the L2/3 and L6 boundaries (Fig. 4c). Many of these neighborhoods involved cell types from multiple layers, even though our permutations kept cell labels of the same layer together. This suggests non-random placement of cell types near layer boundaries. These spatial motifs occurred frequently; on average, 231 cells were assigned to each of the 19 significant spatial motifs. Of note, the L4/5 IT CTX neurons were the only high-resolution cell type to form islands of neurons containing only the same type within 100 um. Our results suggest that by using scProjection to label MERFISH measurements with higher resolution cell type annotations, we can uncover novel spatial neighborhoods of cell types.

### Detection of novel spatial expression patterns of enterocytes in the intestinal epithelium

Because FISH-based RNA measurements are typically limited to measuring expression of a selected gene panel, genome-wide spatial transcriptome sequencing technologies such as Slide-seq^8^, LCM-seq^9^ and Visium by 10x Genomics are often used for more unbiased searches of spatial gene expression patterns. For these spatial transcriptome sequencing technologies, RNA is captured from pre-defined spots of a tissue slice, as opposed to individual transcripts being imaged as in the case of FISH^34^. Unfortunately, each spot therefore potentially contains RNA contributions from more than one cell in close proximity (Fig. 1a), yielding RNA measurements that are not truly single cell but more closely resemble miniature bulk RNA samples^42^. Here we demonstrate that by identifying and extracting the expression profile of the cell type contributing the most RNA to each spot via scProjection, we improve the number of spatial expression patterns detected from these spatial transcriptome sequencing technologies.

We obtained a dataset collected by Moor et al. ^43^ in which they performed LCM-seq on five distinct regions (zones) of the intestinal villus, and also collected a scRNA-seq cell atlas from replicate intestinal villi. They identified spatial expression patterns in the dominant cell type, enterocytes, by (1) identifying marker genes for each zone using the bulk LCM-seq data, (2) assigning zone labels to the scRNA-seq cells using the marker genes, and (3) predicting zone-specific expression through zone-specific averaging of the labeled scRNA-seq data. We reasoned that the identification of marker genes from LCM-seq data could be difficult since each LCM-seq measurement can capture contributions from multiple cells and cell types, thus yielding poor labeling of the single-cell atlas cells. We therefore avoided this critical marker gene selection step by taking the opposite approach: we use scProjection to project the zone-specific LCM-seq samples to the enterocyte single-cell atlas, in order to extract the enterocyte expression patterns within each zone.

**Fig. 5:**
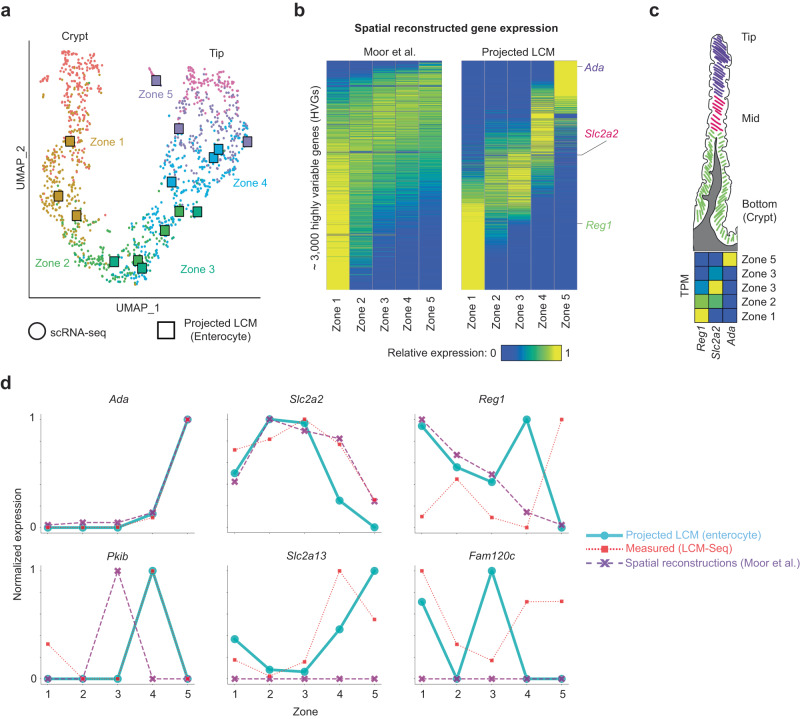
Projection refines spatial expression patterns observed in enterocytes of the intestinal villus. **a** UMAP plot of the single cell atlas (circles) and projected LCM samples (squares) across the zones of the intestinal villus. Single cells are colored based on their zone assignment by Moor et al. **b** Heatmap visualizing the spatial expression patterns of the top 3000 highly variable genes using the spatial inference approach of Moor et al. on the left and after projecting the LCM samples with scProjection on the right. Three marker genes (rows) are labeled: *Ada*, *Slc2a2* and *Reg1*. **c** Schematic of a single intestinal villus, along with the expected dominant expression zone of expression for *Ada*, *Slc2a2* and *Reg1*. Shown below the villus is the measured expression pattern of *Ada*, *Slc2a2* and *Reg1* in the LCM data of the five zones. **d** Line plots comparing the measured and projected expression of top zonated genes across the intestinal villus.

Figure 5a illustrates the projections of the LCM-seq data to the enterocyte single-cell atlas, where the single cells are labeled according to Moor et al. ^43^. The LCM-enterocyte projections are proximal to the single cells assigned to the same zone by Moor et al., suggesting our approach is overall consistent with that of Moor et al. However, our approach identifies 3-fold more zone-specific spatial expression patterns compared to the genes identified by Moor et al. (Fig. 5b). Comparing our predictions of zone-specific genes to smFISH quantifications of the same genes, we successfully called known zone-specific markers such as *Ada*, *Slc2a2*, and *Reg1* as in the original paper, and also identified novel zonated genes such as *Pkib*, *Slc2a13*, and *Fam120c* not correctly captured by the original paper’s approach (Fig. 5c, d). These results suggest RNA projections improve our ability to identify zone-specific expression patterns in dominant cell types such as enterocytes.

### Projection of Patch-seq RNA improves the identification of associations between gene expression and neuron electrophysiology

**Fig. 6:**
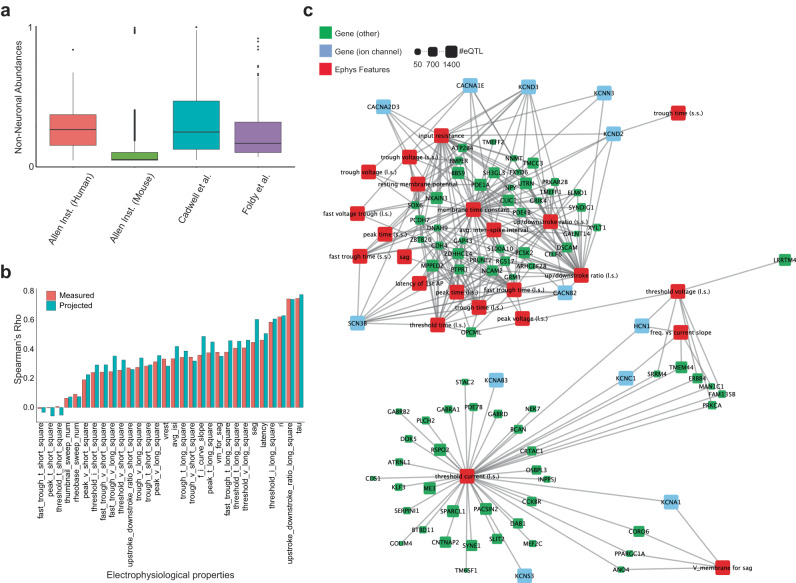
Projection of Patch-seq RNA improves associations identified between gene expression patterns and electrophysiological properties of neurons. **a** Box plot indicates the abundances of non-neuronal (contaminating) RNA estimated by scProjection across all samples of multiple Patch-seq studies (sample size *n* = 273, 3840, 45, and 93 for Allen human, Allen mouse, Cadwell et al., and Foldy et al., respectively). In the box plots, the minima, maxima, centerline, bounds of box, and whiskers represents minimum value in the data, maximum value in the data, median, upper and lower quartiles, and 1.5x interquartile range, respectively. **b** Bar plot indicating the accuracy of gene expression-based prediction of electrophysiology measurements, when predictions are made using either the original measured RNA, or the Sst-projected Patch-seq samples. **c** Gene—electrophysiology correlation network, where edges connect significantly correlated genes and electrophysiology features. Node size is proportional to the number of eQTLs identified in the xQTL study of the ROSMAP cohort (visualization was created using Cytoscape^59^).

Besides spatial transcriptome technologies, there are other single-cell resolution assays that could benefit from scProjection. For example, Patch-seq^10^ is a protocol for collecting RNA, electrophysiological (ephys), and morphological properties from the same neuron, and is critical for linking the molecular and cellular properties of neurons. Patch-seq navigates a micropipette to a neuron of interest in order to simultaneously fill the neuron with a dye, measure its electrophysiological properties and extract its RNA. When applied to in vivo and ex vivo brain slices, the micropipette navigates through many neurons and glia to the neuron of interest. RNA from surrounding neurons and glial often adheres to the micropipette, resulting in the collection of RNA from both the target neuron as well as surrounding cells^12,44,45^. scProjection analysis of several Patch-seq studies indicates cell type abundances from non-neuronal cells are predicted to be as high as 30%, suggesting significant contamination of RNA from non-target cells (Fig. 6a, Fig. S5). We, therefore, hypothesized that projecting Patch-seq RNA measurements to a single cell atlas of neurons before analysis would reduce the effect of contaminating RNA and increase our ability to identify associations between gene expression and electrophysiological measurements of neurons.

We applied scProjection to a set of 4200 Patch-seq measurements targeting mouse GABAergic neurons^41^, together with a reference cell atlas of the mouse brain^5^. Of the 4200 measurements, scProjection predicted that 1912 of them were primarily targeting Sst inhibitory neurons (Fig. S6), consistent with the fact that these 1912 assayed neurons were experimentally identified as Sst before Patch-seq. We focused our experiments on the 1912 predicted Sst inhibitory neurons because they were the best represented type of neuron, and therefore projected the 1912 Patch-seq measurements to the Sst single cells sequenced in the reference atlas.

Here we assumed that more accurate Patch-seq RNA measurements should enable better prediction of ephys properties of neurons from gene expression levels. We found that our RNA projection led to an increase in Spearman correlation from 0.43 to 0.62 on average (*p* = 5 × 10^−18^) when predicting two ephys features, sag and latency (Fig. 6b). On the other hand, the other 28 ephys features were comparably predicted before and after projection. Additionally, we found that our RNA projections identify significant (q < 0.05) cell type-specific associations (correlations) between Sst-projected ion channel gene expression and ephys properties (Fig. S7). These results together suggest that RNA projections remove noise driven by the presence of non-neuronal RNA, which leads to better identification of associations between gene expression and neuron electrophysiology.

Having used scProjection to establish more gene-ephys associations than could be previously appreciated from the original Patch-seq data, we further hypothesized that genetic variation may drive systematic changes in some ephys features, through changes in gene expression patterns. Using cis-eQTLs detected in the human dorsolateral prefrontal cortex^4^, we found that 91 genes’ expression levels were both associated with genetic variation, and also associated with ephys features of neurons. Although gene-ephys associations do not generally imply causation, ion channel genes in particular play direct, critical roles in establishing ephys responses to neuron stimuli. We found 12 ion channels associated with both neuronal firing and under genetic control via cis-SNPs, of which seven of them were only identified via scProjection analysis (and were not identified using the original Patch-seq measurements). We also identified 79 genes not annotated as ion channels but that are associated with both electrophysiology and eQTLs (Fig. 6c), therefore generating additional hypotheses about how SNPs may influence neuron electrophysiology. Overall, our results demonstrate how scProjection pre-processing of Patch-seq RNA samples before analysis can yield additional insight into specific roles of individual ion channels in regulating single neuron electrophysiology, and connect those ephys genes to genetic variation.

## Discussion

In our experiments, we have demonstrated the utility of projections for the extraction of single-cell transcriptomes from diverse single-cell resolution assays such as spatial transcriptomes and Patch-seq. At its heart, projection maps RNA samples into the cell state space defined by a single-cell atlas. Therefore, RNA projections can also potentially play a role in up-sampling the per-cell sequencing depth of spatial and multi-modal sequencing assays, by projecting lower-depth samples into a higher-depth cell atlas. For example, because RNA capture is not per-cell but per-spot for technologies such as Slide-seq, the number of effective transcripts sequenced can vary spot to spot^8^. Furthermore, mRNA capture efficiencies can vary between protocols^46^, and technologies such as SMART-seqv2 yield significantly higher read depth per cell compared to 3’ tagging technologies such as the 10x Chromium^47^. In these scenarios, scProjection could also be used to project true single-cell measurements to another single cell atlas to perform upsampling, even if the original RNA measurements are not from mixed RNA samples.

RNA projections are complementary to deconvolution methods. The goal of deconvolution methods^16–19^ is primarily to estimate the cell type abundances of a set of reference cell populations within a single RNA sample and is a very well-studied problem dating back several decades^48^. While scProjection also computes such cell type abundances for a set of populations, its primary goal is to distinguish intra-cell type variation by also mapping the RNA sample onto the precise cell state within each of the cell type populations that best represents the expression profile of those cell types within the RNA sample. scProjection, therefore, distinguishes intra-cell type variation, whereas deconvolution methods primarily focus on differences in cell type abundances in a sample.

An important feature of scProjection is that it implicitly fits a probability density function (PDF) over the cell state space for each cell type. This is advantageous for several reasons. First, this enables scProjection to reason about the relative frequency of a cell state observed in the training data, where more frequently observed states have higher probability of being projected to. Second, it enables scProjection to interpolate between observed cell states when the training data is small, which can be important for training on rare cell types or on data from smaller studies. Third, scProjection can also naturally ignore outlier observed cells in the training data because they will not appear often in the cell atlas. In contrast, a number of other methods such as CIBERSORTx^16^ either average the expression profiles of all cells of the same type, or map RNA samples to measured single cells in the atlas^23^. Methods that average cells of the same type together will be sensitive to outliers, and more importantly will be unable to account for variation within a given cell type.

One of the caveats of scProjection and related methods, is that by projecting RNA measurements to a reference single-cell atlas, scProjection assumes that the single-cell atlas contains accurate representations of the cell state of cell populations within the RNA sample. There could be scenarios where this is false; for example, projecting RNA from a spatial transcriptome assay of hepatocellular carcinoma samples to a normal liver atlas would miss expression variation in hepatocytes that is driven by carcinomas. Therefore, if no suitable single-cell atlases are publicly available, it would make sense to collect scRNA-seq data on some biological replicate samples in addition to the spatial transcriptome datasets. This experimental design of collecting both scRNA-seq as well as spatial transcriptome data is common^8,30,43,49^ so we expect this caveat to not limit the widespread applicability of scProjection.

Many current computation approaches to infer cell type abundances assume that biological mixtures can be modeled through a series of weighted linear equations. This common modeling approach assumes that all cell types present in the biological mixture are represented in the single-cell reference dataset. scProjection also models the biological mixtures as a weighted linear equation but additionally computes the likelihood of each biological mixture under each cell type model (Figs. S8–S9). Investigation of the likelihood distributions can reveal biological mixtures that are not supported by the single-cell reference, suggesting there may be cell types missing in the single-cell atlas and therefore projection and deconvolution results should be cautiously interpreted (Fig. S3).

Finally, we envision applications of RNA projections beyond what we have illustrated here. For example, databases such as the Gene Expression Omnibus (GEO) catalog gene expression data from bulk RNA samples collected since when RNA sequencing was first deployed. Using the increasing number of single cell atlases derived for different tissues and cell types across organisms, scProjection can be used to re-analyze historic bulk RNA samples to extract average cell states for individual cell populations that contribute to the bulk RNA sample. Cell type-specific changes in case-control studies could then be inferred, as could cell type-specific eQTLs from genetic studies of disease, for example.

## Methods

### scProjection overview

Note our notation follows the convention that scalar variables are italicized, and vectors and matrices are bolded without italics. Our framework, scProjection, projects $N$ gene expression profiles ${{{{{{\bf{b}}}}}}}_{n}\in B$ generated from RNA samples into each of $K$ different cell populations represented in a reference single cell atlas, yielding a new set of gene expression profiles ${{{{{{\bf{x}}}}}}}_{n,k}$, for $k=1,\ldots,K$. scProjection also estimates ${\alpha }_{n,k}$, the proportion of RNA contributed by each cell population $k$ to sample $n$ (Fig. 1). scProjection assumes that each ${{{{{{\bf{b}}}}}}}_{n}$ is a weighted linear combination of the cell population-specific projections ${{{{{{\bf{x}}}}}}}_{n,k}$:

1 $${{{{{{\bf{b}}}}}}}_{n}=\,\mathop{\sum }\limits_{k}^{K}{\alpha }_{n,k}{{{{{{\bf{x}}}}}}}_{n,k}$$

Only ${{{{{{\bf{b}}}}}}}_{n}$ is observed, and the goal is to estimate ${\alpha }_{n,k}$ and ${{{{{{\bf{x}}}}}}}_{n,k}$.

To perform estimation, scProjection leverages a separate reference single cell atlas in which single cells ${{{{{{\bf{s}}}}}}}_{j,k}$ (representing the $j$^th^ cell sequenced for cell population $k$ in the atlas $S$) have been sequenced. In the first step, scProjection trains a deep variational autoencoder (VAE) separately for each cell population $k$ using all single cells sequenced for cell population $k$ $({{{{{{\bf{s}}}}}}}_{*,k})$, yielding a parameter set $\left\{{{{{{{\boldsymbol{\phi }}}}}}}_{k},{{{{{{\boldsymbol{\theta }}}}}}}_{k}\right\}$ (representing the encoder and decoder parameters, respectively) for each cell population $k$. After training, each VAE implicitly defines the set of cell states that projections into cell population $k$ $({{{{{{\bf{x}}}}}}}_{n,k})$ can occupy. In the second step, the VAEs with trained parameters $\{{\hat{{{{{{\boldsymbol{\phi }}}}}}}}_{k}^{(0)},{\hat{{{{{{\boldsymbol{\theta }}}}}}}}_{k}^{(0)}\}$ are used to get initial projections ${\hat{{{{{{\bf{x}}}}}}}}_{n,k}^{(0)}$ by inputting each ${{{{{{\bf{b}}}}}}}_{n}$ into the $k$^th^ VAE and sampling from the output to estimate ${\hat{{{{{{\bf{x}}}}}}}}_{n,k}^{(0)}$. We then estimate the RNA proportions ${\hat{\alpha }}_{n,k}$ by solving Eq. 1 using linear regression by setting ${{{{{{\bf{x}}}}}}}_{n,k}={\hat{{{{{{\bf{x}}}}}}}}_{n,k}^{(0)}$. Finally, in the third step, we fix the mixing proportions ${\hat{\alpha }}_{n,k}$, and re-update all VAE parameters $\left\{{{{{{{\boldsymbol{\phi }}}}}}}_{k},{{{{{{\boldsymbol{\theta }}}}}}}_{k}\right\}$ simultaneously to improve estimates of ${{{{{{\bf{x}}}}}}}_{n,k}$ by maximizing the reconstruction of each ${{{{{{\bf{b}}}}}}}_{n}$.

### scProjection training of cell population-specific VAEs (Step 1)

scProjection uses VAEs to perform the projection of RNA samples ${{{{{{\bf{b}}}}}}}_{n}$ into the gene expression space of each cell population $k$ to yield the projection ${{{{{{\bf{x}}}}}}}_{n,k}$. The set of cell population-specific VAEs are identical in network structure and are comprised of a deep encoder network parameterized by weights ${{{{{{\boldsymbol{\phi }}}}}}}_{k}$, and decoder network parameterized by weights ${{{{{{\boldsymbol{\theta }}}}}}}_{k}$. To train the VAEs, we optimize the following objective function with respect to the VAE parameters $\left\{{{{{{{\boldsymbol{\phi }}}}}}}_{k},{{{{{{\boldsymbol{\theta }}}}}}}_{k}\right\}$, where ${{{{{{\bf{z}}}}}}}_{j,k}$ represents the latent representation of ${{{{{{\bf{s}}}}}}}_{j,k}$:

2 $$L\left(\left\{{{{{{{\boldsymbol{\phi }}}}}}}_{k},{{{{{{\boldsymbol{\theta }}}}}}}_{k}\right\}{{{{{\rm{;}}}}}}\left\{{{{{{{\bf{s}}}}}}}_{j,k}\right\}\right)=\,\mathop{\sum }\limits_{k=1}^{K}\mathop{\sum }\limits_{j=1}^{J}{E}_{{q}_{{{{{{{\boldsymbol{\phi }}}}}}}_{k}}\left({{{{{{\bf{z}}}}}}}_{j,k}\big|{{{{{{\bf{s}}}}}}}_{j,k}\right)}\left[{{\log }}\,{p}_{{{{{{{\boldsymbol{\theta }}}}}}}_{k}}\left({{{{{{\bf{s}}}}}}}_{j,k},|,{{{{{{\bf{z}}}}}}}_{j,k}\right)\right]\\ -\mathop{\prod }\limits_{k=1}^{K}\mathop{\prod }\limits_{j=1}^{J}{D}_{{KL}}\left[{q}_{{{{{{{\boldsymbol{\phi }}}}}}}_{k}}\left({{{{{{\bf{z}}}}}}}_{j,k}\right)\,\left({{{{{{\bf{s}}}}}}}_{j,k}\right){{||}}p\left({{{{{{\bf{z}}}}}}}_{j,k}\right)\right]$$

3 $${q}_{{{{{{{\boldsymbol{\phi }}}}}}}_{k}}\left({{{{{{\bf{z}}}}}}}_{j,k} | {{{{{{\bf{s}}}}}}}_{j,k}\right)=N\left({{{{{{\bf{z}}}}}}}_{j,k}{{{{{\rm{;}}}}}}{\mu }_{{{{{{{\boldsymbol{\phi }}}}}}}_{k}}\left({{{{{{\bf{s}}}}}}}_{j,k}\right),\,{\sigma }_{{{{{{{\boldsymbol{\phi }}}}}}}_{k}}^{2}\left({{{{{{\bf{s}}}}}}}_{j,k}\right)I\right)$$

4 $${p}_{{{{{{{\boldsymbol{\theta }}}}}}}_{k}}\left({{{{{{\bf{s}}}}}}}_{j,k}|{{{{{{\bf{z}}}}}}}_{j,k}\right)=N\left({{{{{{\bf{s}}}}}}}_{j,k}{{{{{\rm{;}}}}}}{\mu }_{{{{{{{\boldsymbol{\theta }}}}}}}_{k}}\left({{{{{{\bf{z}}}}}}}_{j,k}\right),\,{\sigma }_{{{{{{{\boldsymbol{\theta }}}}}}}_{k}}^{2}\left({{{{{{\bf{z}}}}}}}_{j,k}\right)I\right)$$

The functions $\left\{{\mu }_{{{{{{{\boldsymbol{\phi }}}}}}}_{k}}\left(\cdot \right),{\sigma }_{{{{{{{\boldsymbol{\phi }}}}}}}_{k}}^{2}\left(\cdot \right)\right\}$ and $\left\{{\mu }_{{{{{{{\boldsymbol{\theta }}}}}}}_{k}}\left(\cdot \right),{\sigma }_{{{{{{{\boldsymbol{\theta }}}}}}}_{k}}^{2}\left(\cdot \right)\right\}$ represent the mean and variance of the normal distribution predicted by the encoder and decoder, respectively. The parameters of the VAEs $\left\{{{{{{{\boldsymbol{\phi }}}}}}}_{k},{{{{{{\boldsymbol{\theta }}}}}}}_{k}\right\}$ are regularized through 30% dropout^50^, batch normalization^51^ and L2 weight regularization to ensure robust training. ADAM^52^ is used for optimization with a decaying learning rate starting at 10^−3^ and a smooth warmup of the KL term in the ELBO, which has been shown to produce more accurate reconstructions^53^. We denote the trained VAE parameters by $\{{\hat{{{{{{\boldsymbol{\phi }}}}}}}}_{k}^{(0)},{\hat{{{{{{\boldsymbol{\theta }}}}}}}}_{k}^{(0)}\}.$

For the experiments in which we infer genome-wide expression measurements from limited sets of marker genes such as those measured by MERFISH, the structure of the VAE can be adjusted to be asymmetric with the input measurements to the encoder defined by a subset of gene expression measurements ${G}_{e}\subseteq G$ (corresponding to marker genes). The decoder output is still defined by the full set of gene expression measurements $G$ made in the single cell atlas. Only estimates of those genes ${G}_{e}$ directly measured in mixture samples ${{{{{{\bf{b}}}}}}}_{n}$ are used in subsequent steps of scProjection.

### scProjection estimation of cell type abundance of each cell population (Step 2)

Here, scProjection projects each RNA sample ${{{{{{\bf{b}}}}}}}_{n}$ to each cell population $k$ via the VAE parameterized by $\{{\hat{{{{{{\boldsymbol{\phi }}}}}}}}_{k}^{(0)},{\hat{{{{{{\boldsymbol{\theta }}}}}}}}_{k}^{(0)}\}$ to estimate ${\hat{{{{{{\bf{x}}}}}}}}_{n,k}^{(0)}$, as well as the predicted variance over those estimates:

5 $${\hat{{{{{{\bf{x}}}}}}}}_{n,k}^{\left(0\right)}\,=\,{\mu }_{{\hat{{{{{{\boldsymbol{\theta }}}}}}}}_{k}^{\left(0\right)}}\left({\mu }_{{\hat{{{{{{\boldsymbol{\phi }}}}}}}}_{k}^{\left(0\right)}}\left({{{{{{\bf{b}}}}}}}_{n}\right)\right)$$

6 $$\,{\hat{\sigma }}_{n,k}^{2}={\sigma }_{{\hat{{{{{{\boldsymbol{\theta }}}}}}}}_{k}^{\left(0\right)}}^{2}\left({\mu }_{{\hat{{{{{{\boldsymbol{\phi }}}}}}}}_{k}^{\left(0\right)}}\left({{{{{{\bf{b}}}}}}}_{n}\right)\right)$$

Then, we estimate the mixture proportions ${\alpha }_{n,k}$ and nuisance parameters of a multi-layer perceptron ${f}_{{\sigma }_{b}}$ (and hold all other variables fixed) by optimizing the following objective function:

7 $$L\left({{{{{{\bf{b}}}}}}}_{n}\right)=\mathop{\sum }\limits_{n=1}^{N}{{\log }}\,N({{{{{{\bf{b}}}}}}}_{n}|\mathop{\sum }\limits_{k}^{K}{\hat{{{{{{\bf{x}}}}}}}}_{j,k}^{(0)}{\alpha }_{n,k},\,{f}_{{\sigma }_{b}}({\hat{{{{{{\boldsymbol{\sigma }}}}}}}}_{n,:}^{2}\oplus {{{{{{\boldsymbol{\alpha }}}}}}}_{n,:})I)$$

Where $\oplus$ is the vector concatenation operator. Optimization is performed with ADAM^52^ and a learning rate of 10^−3^ until convergence. The estimated mixing proportions ${\hat{\alpha }}_{n,k}$ are kept fixed for the remainder of the training procedure.

### scProjection final estimates of RNA projections (Step 3)

In this step, scProjection re-optimizes the encoders and decoders of the individual VAEs $\left\{{{{{{{\boldsymbol{\phi }}}}}}}_{k},{{{{{{\boldsymbol{\theta }}}}}}}_{k}\right\}$ by minimizing the following composite objective function, which includes the likelihood of both the single cell atlas data ${{{{{{\bf{s}}}}}}}_{j,k}$ and the RNA samples ${{{{{{\bf{b}}}}}}}_{n}$, and where ${\sigma }_{n,k}^{2}={\sigma }_{{{{{{{\boldsymbol{\theta }}}}}}}_{k}}^{2}({\mu }_{{{{{{{\boldsymbol{\phi }}}}}}}_{k}}({{{{{{\bf{b}}}}}}}_{n}))$:

8 $${{\mbox{ELBO}}}=	\,\mathop{\sum }\limits_{n}^{B}{{\log }}\,N\left({{{{{{\bf{b}}}}}}}_{n}|\mathop{\sum }\limits_{k=1}^{K}{\mu }_{{{{{{{\boldsymbol{\theta }}}}}}}_{k}}\left({\mu }_{{{{{{{\boldsymbol{\phi }}}}}}}_{k}}\left({{{{{{\bf{b}}}}}}}_{n}\right)\right){\hat{\alpha }}_{n,k},\,{f}_{{\sigma }_{b}}\left({{{{{{\boldsymbol{\sigma }}}}}}}_{n,:}^{2}\oplus {\hat{{{{{{\boldsymbol{\alpha }}}}}}}}_{n,:}\right)I\right)\, \\ 	+ \mathop{\sum}\limits_{k}\,\mathop{\sum}\limits_{j}{E}_{{q}_{{{{{{{\boldsymbol{\phi }}}}}}}_{k}}\left({{{{{{\bf{z}}}}}}}_{j,k}|{{{{{{\bf{s}}}}}}}_{j,k}\right)}\left[\,{{\log }}\,{p}_{{{{{{{\boldsymbol{\theta }}}}}}}_{k}}\left({{{{{{\bf{s}}}}}}}_{j,k},|,{{{{{{\bf{z}}}}}}}_{j,k}\right)\,\right]\,\\ 	-\left[\mathop{\sum}\limits_{k}\,\mathop{\sum}\limits_{n}{D}_{{KL}}\left({q}_{{{{{{{\boldsymbol{\phi }}}}}}}_{k}}\left({{{{{{\bf{z}}}}}}}_{n,k}|{{{{{{\bf{b}}}}}}}_{n}\right)\right){||}\,p\left({{{{{{\bf{z}}}}}}}_{n,k}\right)\right] \\ 	+ \mathop{\sum}\limits_{k}\,\mathop{\sum}\limits_{j}\,{D}_{{KL}}\left[{q}_{{{{{{{\boldsymbol{\phi }}}}}}}_{k}}\left({{{{{{\bf{z}}}}}}}_{j,k}|{{{{{{\bf{s}}}}}}}_{j,k}\right){||}\,p\left({{{{{{\bf{z}}}}}}}_{j,k}\right)\right]$$

Note in this case, the VAE parameters are initially set to ${{{{{{\boldsymbol{\phi }}}}}}}_{k}={\hat{{{{{{\boldsymbol{\phi }}}}}}}}_{k}^{(0)}$ and ${{{{{{\boldsymbol{\theta }}}}}}}_{k}={\hat{{{{{{\boldsymbol{\theta }}}}}}}}_{k}^{(0)}$ before optimization, and the parameters of ${f}_{{\sigma }_{b}}$ are fixed at their values estimated at Step 2. Intuitively, we are adjusting the RNA projections ${{{{{{\bf{x}}}}}}}_{n,k}={\mu}_{{{{{{{\boldsymbol{\theta}}}}}}}_{k}}\left({\mu }_{{{{{{{\boldsymbol{\phi}}}}}}}_{k}}\left({{{{{{\bf{b}}}}}}}_{n}\right)\right)$ to better predict the RNA sample ${{{{{{\bf{b}}}}}}}_{n}$, because the single cell reference data may be collected in a different experiment from the RNA samples. The single cell data are included in the objective function and serve as a regularization term to ensure identifiability of each VAE as specific to one cell population $k$. After training to obtain final VAE parameter estimates $\{{\hat{{{{{{\boldsymbol{\phi }}}}}}}}_{k}^{(1)},{\hat{{{{{{\boldsymbol{\theta }}}}}}}}_{k}^{(1)}\}$, we estimate our final RNA projections ${\hat{{{{{{\bf{x}}}}}}}}_{n,k}={\mu }_{{\hat{{{{{{\boldsymbol{\theta }}}}}}}}_{k}^{(1)}}({\mu }_{{\hat{{{{{{\boldsymbol{\phi }}}}}}}}_{k}^{(1)}}({{{{{{\bf{b}}}}}}}_{n})).$

### Acquisition and preprocessing of the intestinal villus dataset

We obtained the gene expression matrices for the LCM-seq, scRNA-seq and spatial reconstructions experiments described in Moor et al. ^43^ from GSE109413 and 10.5281/zenodo.1320734. We independently normalized the count matrices, then scaled and centered using Seurat’s (v4) NormalizeData (without log transform) and ScaleData functions. We retained the union of the marker genes of each cell type identified from the original study, together with the top 2000 variable genes of both LCM-seq and scRNA-seq.

### Acquisition and preprocessing of the brain MERFISH dataset

We obtained the processed MERFISH gene luminescence matrix described in Moffitt et al. ^26^ from dryad.8t8s248 and the scRNA-seq count matrix from GSE113576. We independently preprocessed each data modality by using Seurat’s NormalizeData and ScaleData functions. We removed entire cell types from the scRNA-seq data that had no analog in the MERFISH experiments and are defined in Table S9 of the original study^26^. We retained the union of the marker genes of each cell type identified in the original study, together with the top 2000 variable genes across the entire scRNA-seq atlas.

### Acquisition and preprocessing of the mouse Patch-seq dataset

We obtained the gene count matrix for the mouse Patch-seq experiments described in Berg et al.^54^ from https://portal.brain-map.org/explore/classes/multimodal-characterization on January 2019. We discarded samples that did not pass QC as defined in the original paper in both the RNA and electrophysiology modalities. We normalized the count matrix to relative counts (RC) (without log transform), then scaled and centered using Seurat’s NormalizeData and ScaleData functions. We retained the union of the marker genes of each cell type identified from the original study, together with the top 2000 variable genes across each of the cell types defined in the snRNA-seq.

### Acquisition and preprocessing of the mouse brain atlas

We obtained the gene count matrix for the mouse brain atlas described in Yao et al. ^5^ from the Allen Institute Cell Types database: RNA-Seq data page on the Allen Institute’s webpage. We normalized the count matrix, then scaled and centered using Seurat’s NormalizeData and ScaleData functions. We retained the union of the marker genes of each cell type reported in the original study, together with the top 2000 variable genes across each of the cell types defined in the snRNA-seq.

### Acquisition and preprocessing of the Tasic et al. mouse brain atlas

We obtained the gene count matrix for the mouse brain atlas described in Tasic et al. from the Allen Institute Cell Types database: RNA-Seq data page on the Allen Institute’s webpage. We normalized the count matrix, then scaled and centered using Seurat’s NormalizeData and ScaleData functions. We retained the union of the marker genes of each cell type reported in the original study, together with the top 2000 variable genes across each of the cell types defined in the snRNA-seq.

### Acquisition and preprocessing of the CellBench benchmark

We obtained the gene count matrix for the RNA mixture experiments in CellBench described in Tian et al.^24^ from the R data file mRNAmix_qc.RData available on GitHub (https://github.com/Shians/CellBench). We normalized the count matrix to RC (without log transform), then scaled and centered using Seurat’s NormalizeData and ScaleData functions. We retained the union of the marker genes of each cell type identified by CIBERSORTx, together with the top 3000 variable genes computed separately on the RNA mixtures profiled on CEL-Seq2 and SORT-Seq.

### Acquisition and preprocessing of the ROSMAP-IHC benchmark

We obtained the gene count matrix for the bulk-RNA experiments and IHC measurements described in Patrick et al. from the R data files available on GitHub (https://github.com/ellispatrick/CortexCellDeconv). We normalized the count matrix to RC (without log transform), then scaled and centered using Seurat’s NormalizeData and ScaleData functions. We retained the union of the marker genes of each cell type reported in Darmanis et al. ^55^, together with the top 2000 variable genes.

### Execution of deconvolution methods

In the two sections below on benchmarking deconvolution (cell proportion estimation) methods in different datasets, we compared scProjection against CIBERSORTx^16^, MuSiC^17^, NNLS, dtangle^18^, DSA^19^, and single gene deconvolution. Each method was run based on method-specific guidelines provided by the original authors and following the workflows defined by in tutorials for each approach. Prior to running each method, the FindVariableFeatures function implemented in Seurat was used to identify the most variable genes for a consistent subsetting of the data matrices. CIBERSORTx was provided counts for all highly variable genes in the scRNA-seq data along with cell type annotations to create a signature matrix. Then counts for all highly variable genes in the mixture data were provided to CIBERSORTx which then estimates RNA proportions. MuSiC was provided counts for all highly variable genes in the scRNA-seq and mixture data along with cell type annotations. NNLS (as implemented by us in R) was provided the TPM values for all highly variable genes in the scRNA-seq and mixture data. Proportions from NNLS for cell type ${k}$ were computed by summing the learned weights across all cells annotated as cell type $k$; this was repeated for each cell type and each mixture sample. dtangle was provided with a mean count vector per cell type in the scRNA-seq data and the original counts from the mixture data along with cell type markers and annotations. DSA was provided with the original counts for the mixture data and cell type specific marker genes. Single gene deconvolution was performed by identifying individual marker genes of each cell type, which were used to estimate the relative proportion of each cell type with respect to the remaining markers.

### Benchmarking cell population proportion estimation on the CellBench dataset

The CellBench dataset provides gene expression profiles obtained from sequencing titrated RNA mixtures from three human lung adenocarcinoma cell lines (H1975, H2228, HCC827), as well as single cell RNA profiles from each cell line. Sequencing was performed using either plate based (CEL-Seq2 or Drop-Seq) or droplet based (10x Chromium and Drop-seq Dolomite) protocols. The proportion of RNA from each cell line was recorded for each mixture and defines a baseline for methods aiming to computationally estimate the cell type abundances. We trained scProjection using the RNA mixtures as inputs ${{{{{{\bf{b}}}}}}}_{n}$ and the single cell data as the atlas *S*. We treated the scProjection estimates ${\hat{\alpha }}_{n,k}$ as our predictions of abundances for each cell type. We then compared scProjection-based deconvolution against other methods as described above (Fig. S2).

### Benchmarking cell population proportion estimation on the ROSMAP-IHC dataset

To provide a more challenging and realistic deconvolution benchmark, we used the ROSMAP-IHC dataset consisting of 70 bulk RNA samples from the dorsolateral prefrontal cortex (DLPFC), an scRNA-seq atlas derived from the DLPFC, and cell type abundances estimated using IHC from adjacent samples to those samples used for sequencing. The bulk RNA, reference single cell atlas and cell type abundances were collected and estimated in three different studies, thus introducing technical and biological variability between data modalities that does not exist in the CellBench study. We trained scProjection using the RNA mixtures as inputs ${{{{{{\bf{b}}}}}}}_{n}$ and the single cell data as the atlas *S*. We treated the scProjection estimates ${\hat{\alpha }}_{n,k}$ as our predictions of abundances for each cell type. We then compared scProjection-based deconvolution against other methods as described above (Fig. 1B). Furthermore, for each proportion estimated by scProjection, we assign a confidence score indicating the certainty of the mixture being assigned to a specific cell type (Fig. S8).

### Prediction of cell population using scProjection

From scProjection’s estimates of cell population specific abundances, treated as probabilistic class assignments, the class with maximal probability is assigned as the cell population label for each sample.

### Cell annotation with k-NN label transfer

We label RNA mixtures using k-NN based on the labels of the neighboring cells in the single cell atlas the mixture was projected into.

### Zonated gene expression scoring

For each gene, we compute the distance from an idealized zone-specific measurement as the difference between ${gen}{e}_{{ideal}}\,$= (1,0,0,0,0) and the computed gene zonation score vector. A threshold was set based on the 75^th^ quantile of the resulting scores to compare the number of zonated genes across methods.

### Benchmarking cell population proportion estimation on the MERFISH dataset

The MERFISH dataset from Moffitt et al. provides the gene expression profiles of MERFISH samples as well as single cells from the same regions of mouse brain. Animal 1, which had the largest sample size in MERFISH data, along with the scRNA-seq reference data from the same study were selected. The features were matched by intersecting the gene set measured in MERFISH data and the top 2000 highly variable genes from scRNA-seq data called using Seurat’s FindVariableFeatures function, and we retained 115 genes for the cell type abundance estimation experiments. TP10K (without log transform) normalization and z-score centering and scaling were applied to the count matrix for both datasets using Seurat’s NormalizeData and ScaleData functions. We trained scProjection using the MERFISH samples as inputs ${{{{{{\bf{b}}}}}}}_{n}$ and the single cell data as the atlas *S*. We treated the scProjection estimates ${\hat{\alpha }}_{n,k}$ as our predictions of abundances for each cell type. We then compared scProjection-based deconvolution against other methods including both Tangram “cell” and “cluster” modes (https://github.com/broadinstitute/Tangram), and SpatialDWLS (wrapped in the Giotto: https://github.com/RubD/Giotto/, a spatial transcriptomics data analysis toolbox).

### Summarizing the cell population proportion estimation on MERFISH dataset

The deconvolution results from each of scProjection, Tangram, and SpatialDWLS included the sample by cell type annotation matrix reflecting the estimates of cell type abundances of each MERFISH sample. All samples from the same cell type label defined in the original Moffitt et al. study were grouped together to calculate the cell-type-specific abundance estimations by averaging across samples.

### Simulating multi-cell mixed RNA samples for projection performance benchmarking

We simulated 4 sets of pseudo-bulk mixed RNA samples by combining single cells from up to eight neuronal subclasses (L2/3 IT CTX, L4/5 IT CTX, L5 IT CTX, L6 IT CTX, Lamp5, Pvalb, Sst, and Vip) using the mouse Primary Motor Area (MOp) scRNA-seq data from the Cell Types Database of the Allen Brain Map (https://celltypes.brain-map.org/api/v2/well_known_file_download/738607155). The 4 sets of simulated mixed RNA samples were comprised of mixtures of either two (L2/3 IT and Lamp5), four (L2/3 IT CTX, L5 IT CTX, Lamp5, and Sst), six (L2/3 IT CTX, L5 IT CTX, L6 IT CTX, Lamp5, Sst, and Vip), or eight (L2/3 IT CTX, L4/5 IT CTX, L5 IT CTX, L6 IT CTX, Lamp5, Pvalb, Sst, and Vip) different neuronal subclasses, respectively. We varied the number of contributing cell types in order to ensure representation of RNA mixtures of varying levels of complexity of cell type composition. Relative counts (with log transformation and library size factor of 10,000) normalization was applied to the raw count matrix using Seurat’s NormalizeData function, and the top 2000 highly variable genes were selected using the Seurat’s FindVaribleFeatures function. Then each of the 4 different mixed RNA sample sets were generated by randomly sampling 5000 single cells from each of the component cell subclasses of the mouse MOp dataset, followed by summing the transcriptional profile of each single cell from each subclass to build 5000 mixed RNA samples for each sample set. In total, we generated 20,000 mixed RNA samples.

To estimate scProjection performance under realistic conditions, we projected the simulated mixed RNA samples into an independent primary visual cortex (VISp) scRNA-seq cell atlas^5^ that contained a comparable set of neuronal subclasses as that used to generate the mixed RNA samples. To establish baseline projection performance, we compared scProjection against two other methods, Tangram^23^ and uniPort^38^, which also can project mixed RNA samples to a reference single cell atlas. We followed the training procedures outlined in the tutorials of Tangram and uniPort for projection (https://tangram-sc.readthedocs.io/en/latest/tutorial_sq_link.html; https://uniport.readthedocs.io/en/latest/examples/PDAC/pdac.html), respectively.

To quantify projection performance, four metrics adapted from Li et al. ^27^ and Singh et al. ^56^ were used to evaluate the performance of each method. They are correlation coefficient (CC), root-mean-square error (RMSE), Jensen-Shannon distance (JSD), and the “fraction of samples closer than the true match” (FOSCTTM). Figure S10A demonstrates these metrics are broadly correlated with each other as expected. We used these performance metrics as follows. For each mixed RNA sample ${{{{{{\bf{b}}}}}}}_{n}$, it was simulated by combining one observed single cell from each of $K$ populations (where $K\in \left\{{{{{\mathrm{2,4,6,8}}}}}\right\}$), denoted as ${{{{{{\bf{s}}}}}}}_{n,1}$, …, ${{{{{{\bf{s}}}}}}}_{n,K}$. Each sample ${{{{{{\bf{b}}}}}}}_{n}$ was projected to $K$ different cell populations, yielding ${\hat{{{{{{\bf{x}}}}}}}}_{n,k}$, using one of the projection methods. We treat ${\hat{{{{{{\bf{x}}}}}}}}_{n,k}$ as the computational estimate of ${{{{{{\bf{s}}}}}}}_{n,k}$, and therefore compute the four performance metrics by comparing ${\hat{{{{{{\bf{x}}}}}}}}_{n,k}$ to ${{{{{{\bf{s}}}}}}}_{n,k}$ as follows (let $T$ denote the indices of all mixed RNA samples in the held-out test set, and $G$ denote the number of genes):

9 $${{{{{\rm{CC}}}}}}=\frac{1}{\left|T\right|}\mathop{\sum}\limits_{n\in T}{{{{{\rm{Spearman}}}}}}\left({\hat{{{{{{\bf{x}}}}}}}}_{n,k},{{{{{{\bf{s}}}}}}}_{n,k}\right)$$

10 $${{{{{\rm{RMSE}}}}}}=\,\frac{1}{\left|T\right|}\mathop{\sum}\limits_{n\in T}\sqrt{\frac{{\|{\hat{{{{{{\bf{x}}}}}}}}_{n,k}-{{{{{{\bf{s}}}}}}}_{n,k}\|}_{2}^{2}}{G}}$$

11 $${{{{{\rm{JSD}}}}}}=\,\frac{1}{\left|T\right|}\mathop{\sum}\limits_{n\in T}\sqrt{\frac{1}{2}D\left({\hat{{{{{{\bf{x}}}}}}}}_{n,k}\parallel {{{{{{\bf{s}}}}}}}_{n,k}\right)+\frac{1}{2}D\left({{{{{{\bf{s}}}}}}}_{n,k}\parallel {\hat{{{{{{\bf{x}}}}}}}}_{n,k}\right)}$$

12 $${{{{{\rm{FOSCTTM}}}}}}=\frac{1}{{|T|}}{\sum }_{n\in T}\left(\frac{1}{\left|T\right|-1}{\sum }_{m\in T,m\ne n}\left[{\|{\hat{{{{{{\bf{x}}}}}}}}_{m,k}-{{{{{{\bf{s}}}}}}}_{n,k}\|}_{2}^{2} < {\|{\hat{{{{{{\bf{x}}}}}}}}_{m,k}-{{{{{{\bf{s}}}}}}}_{n,k}\|}_{2}^{2}\right]\right)$$

Where ${{\mbox{Spearman}}}\left(\cdot \right)$ is the Spearman’s rank correlation coefficient, $D\left(\cdot \parallel \cdot \right)$ is the Kullback-Leibler divergence, and $\left[\cdot \right]$ is the indicator function that returns $1$ if true, else $0$. Higher CC values and lower RMSE, JSD, and FOSCTTM values indicate better projection performance. Additionally, similar to Li et al. ^27^, we defined a “relative projection performance score” by aggregating the ranks of CC, RMSE, JSD, and FOSCTTM as a summary metric to compare the projection performance of each method. More specifically, we rank all methods in increasing order of CC, and decreasing order of RMSE, JSD, and FOSCTTM. We then assign each method a final average rank that represents their composite score, based on the four individual metric rankings.

### Imputing spatial transcripts distribution on osmFISH dataset

To evaluate the performance of imputing the spatial distribution of transcripts that are not measured in the spatial transcriptomics datasets, we conducted systemic benchmarking experiments on a gold standard mouse somatosensory cortex osmFISH dataset from the Linnarsson Lab^29^ and leveraged a scRNA-seq dataset containing 3005 mouse cortex cells^35 ^as the paired reference. We imported the datasets via a scvi-tools built-in data function^57^.

There are 33 genes measured in the spatial osmFISH dataset in total, and we applied leave-one-gene-out procedure to test the imputation performance on each gene individually using scProjection, uniPort^38^, Tangram^23^, gimVI^36^, and SpaGE^37^, using the same evaluation metric as developed previously^27^. Briefly, we adapted the evaluation metrics from the benchmarking paper Li et al.^27^ including Spearman’s correlation coefficient (SCC), structural similarity index measure (SSIM), root means square error (RMSE), Jensen-Shannon distance (JSD), as well as the overall relative imputation performance score that is based on the average ranking of the 4 metrics. The SCC, RMSE, and JSD metrics are similar to what have been described above but were used to assess the accuracy between the ground truth gene expression and imputed patterns across spots on the held-out test set of spatial data. Figure S10B demonstrates these metrics are broadly correlated with each other as expected. The SSIM value was calculated by first transforming the expression value to the range between 0 and 1 and then applied the structural similarity calculation^27^. For each gene, higher SSIM values represent better imputation accuracy.

We first sorted the SCC and SSIM values of each method in ascending order, and then sorted RMSE and JSD values individually in descending order to get their corresponding ranks. Each method was assigned a final rank based on its average rank across the four metrics (SCC, SSIM, RMSE, JSD). We replaced the Pearson’s correlation coefficient (PCC) used originally in Li et al. ^27^ by Spearman’s correlation coefficient (SCC) because SCC was used as a metric for imputation performance in most tutorials of the methods tested in this gene imputation benchmark experiment^36–38^.

We followed the guidelines in these tutorials for executing the competing imputation methods:

- Tangram: https://tangram-sc.readthedocs.io/en/latest/tutorial_link.html.
- gimVI: https://docs.scvi-tools.org/en/stable/tutorials/notebooks/gimvi_tutorial.html.
- SpaGE: https://github.com/tabdelaal/SpaGE/blob/master/SpaGE_Tutorial.ipynb.
- uniPort: https://uniport.readthedocs.io/en/latest/examples/MERFISH/MERFISH_impute.html.

### Quantifying contamination in Patch-seq RNA measurements

To determine the amount of non-neuronal contamination for each Patch-seq sample, we take the sum of abundances across all non-neuronal cell types (microglia, endothelial, oligodendrocyte, oligodendrocyte precursor cells, astrocytes) as the percent contamination per sample.

### Statistics & reproducibility

All statistical calculations were implemented in Python (v3.8.13; https://www.python.org) or R (v3.6.1 or v4.0.1; https://cran.r-project.org). The detailed statistical tests were indicated in corresponding figure legends where applicable. No statistical method was used to predetermine sample size. No data were excluded from the analyses. The experiments were not randomized. This study does not involve group allocation that requires blinding.

### Reporting summary

Further information on research design is available in the Nature Portfolio Reporting Summary linked to this article.

### Supplementary information

Supplementary Information

Reporting Summary

### Source data

Source Data

## Data Availability

All data analyzed in this article are publicly available through online sources. The gene count matrix for the RNA mixture experiments in CellBench^24^ is provided in the R data file that is available at https://github.com/Shians/CellBench. The gene count matrix of the bulk-RNA experiments and IHC measurements for the ROSMAP-IHC benchmark can be found at https://github.com/ellispatrick/CortexCellDeconv. “Mouse Primary Motor Area (MOp) [https://portal.brain-map.org/atlases-and-data/rnaseq/mouse-aca-and-mop-smart-seq]” and “Mouse Primary Visual Cortex (VISp) [https://portal.brain-map.org/atlases-and-data/rnaseq/mouse-v1-and-alm-smart-seq]” scRNA-seq datasets are from the Cell Types Database of the Allen Brain Map. We obtained the gene count matrix for the mouse brain atlas described in Yao et al. ^5^ and Tasic et al. from the Allen Institute Cell Types database: RNA-Seq data page on the Allen Institute’s webpage (https://portal.brain-map.org/atlases-and-data/rnaseq). The MERFISH gene luminescence matrix described in Moffitt et al. ^26^ can be accessed from “DRYAD [10.5061/dryad.8t8s248]” and the scRNA-seq count matrix can be found at NCBI Gene Expression Omnibus (GEO) database with accession ID: “GSE113576”. The gene expression matrices for the LCM-seq, scRNA-seq and spatial reconstructions experiments described in Moor et al. ^43^ can be found at NCBI GEO database with accession ID: “GSE109413” and Zenodo open repository 10.5281/zenodo.1320734. The gene count matrices for mouse and human cortex Patch-seq experiments are available at https://portal.brain-map.org/explore/classes/multimodal-characterization, and the paired electrophysiological recording datasets are available at The DANDI Archive site with ID: “000020” and “000023”, respectively. The gene count matrices of Patch-seq studies from the Foldy et al. and Cadwell et al. have been deposited at NCBI GEO database with accession ID: “GSE75386”, and “E-MTAB-4092” respectively. All other data supporting the findings of this study are available within the article and its supplementary files. Any additional requests for information can be directed to, and will be fulfilled by, the lead contact. Source data are provided as a Source Data file. Source data are provided with this paper.

## Code availability

The scProjection framework was implemented in the ‘scProjection’ Python package, which can be installed through PyPI (https://pypi.org/project/scProjection/), and the code is available at https://github.com/quon-titative-biology/scProjection^58^ along with tutorials.

## References

[1] Hodge RD (2019). Conserved cell types with divergent features in human versus mouse cortex. Nature.

[2] Tasic B (2018). Shared and distinct transcriptomic cell types across neocortical areas. Nature.

[3] Tabula Muris Consortium. (2018). Single-cell transcriptomics of 20 mouse organs creates a Tabula Muris. Nature.

[4] Mathys H (2019). Single-cell transcriptomic analysis of Alzheimer’s disease. Nature.

[5] Yao Z (2021). A taxonomy of transcriptomic cell types across the isocortex and hippocampal formation. Cell.

[6] Travaglini KJ (2020). A molecular cell atlas of the human lung from single-cell RNA sequencing. Nature.

[7] Bakken TE (2021). Comparative cellular analysis of motor cortex in human, marmoset and mouse. Nature.

[8] Rodriques SG (2019). Slide-seq: a scalable technology for measuring genome-wide expression at high spatial resolution. Science.

[9] Espina V (2006). Laser-capture microdissection. Nat. Protoc..

[10] Cadwell CR (2016). Electrophysiological, transcriptomic and morphologic profiling of single neurons using Patch-seq. Nat. Biotechnol..

[11] Földy C (2016). Single-cell RNAseq reveals cell adhesion molecule profiles in electrophysiologically defined neurons. Proc. Natl. Acad. Sci. USA..

[12] Tripathy SJ (2018). Assessing transcriptome quality in patch-seq datasets. Front Mol Neurosci.

[13] Xia C, Fan J, Emanuel G, Hao J, Zhuang X (2019). Spatial transcriptome profiling by MERFISH reveals subcellular RNA compartmentalization and cell cycle-dependent gene expression. PNAS.

[14] Kingma, D. P. & Welling, M. Auto-Encoding Variational Bayes. Auto-Encoding Variational Bayes. In *Proceedings of the International Conference on Learning Representations (ICLR).**arXiv:1312.6114 [cs, stat]* (2014).

[15] Mohammadi S, Davila-Velderrain J, Kellis M (2020). A multiresolution framework to characterize single-cell state landscapes. Nat. Commun..

[16] Newman AM (2019). Determining cell type abundance and expression from bulk tissues with digital cytometry. Nat. Biotechnol..

[17] Wang X, Park J, Susztak K, Zhang NR, Li M (2019). Bulk tissue cell type deconvolution with multi-subject single-cell expression reference. Nat. Commun..

[18] Hunt GJ, Freytag S, Bahlo M, Gagnon-Bartsch J (2019). A. dtangle: accurate and robust cell type deconvolution. Bioinformatics.

[19] Zhong Y, Wan Y-W, Pang K, Chow LM, Liu Z (2013). Digital sorting of complex tissues for cell type-specific gene expression profiles. BMC Bioinformatics.

[20] Stokkum, K. M. M. and I. H. M. van. nnls: The Lawson-Hanson algorithm for non-negative least squares (NNLS). (2012).

[21] Dries R (2021). Giotto: a toolbox for integrative analysis and visualization of spatial expression data. Genome Biol..

[22] Dong R, Yuan G-C (2021). SpatialDWLS: accurate deconvolution of spatial transcriptomic data. Genome Biol..

[23] Biancalani T (2021). Deep learning and alignment of spatially resolved single-cell transcriptomes with Tangram. Nat. Methods.

[24] Tian L (2019). Benchmarking single cell RNA-sequencing analysis pipelines using mixture control experiments. Nat. Methods.

[25] Bennett DA (2018). Religious orders study and rush memory and aging project. JAD.

[26] Moffitt JR (2018). Molecular, spatial, and functional single-cell profiling of the hypothalamic preoptic region. Science.

[27] Li B (2022). Benchmarking spatial and single-cell transcriptomics integration methods for transcript distribution prediction and cell type deconvolution. Nat. Methods.

[28] Chen KH, Boettiger AN, Moffitt JR, Wang S, Zhuang X (2015). Spatially resolved, highly multiplexed RNA profiling in single cells. Science.

[29] Codeluppi S (2018). Spatial organization of the somatosensory cortex revealed by osmFISH. Nat. Methods.

[30] Zhang M (2021). Spatially resolved cell atlas of the mouse primary motor cortex by MERFISH. Nature.

[31] Borm LE (2022). Scalable in situ single-cell profiling by electrophoretic capture of mRNA using EEL FISH. Nat. Biotechnol..

[32] Lu Y (2021). Spatial transcriptome profiling by MERFISH reveals fetal liver hematopoietic stem cell niche architecture. Cell Discov..

[33] Fang R (2022). Conservation and divergence of cortical cell organization in human and mouse revealed by MERFISH. Science.

[34] Williams CG, Lee HJ, Asatsuma T, Vento-Tormo R, Haque A (2022). An introduction to spatial transcriptomics for biomedical research. Genome Med.

[35] Zeisel A (2015). Cell types in the mouse cortex and hippocampus revealed by single-cell RNA-seq. Science.

[36] Lopez, R. et al. A joint model of unpaired data from scRNA-seq and spatial transcriptomics for imputing missing gene expression measurements. Preprint at http://arxiv.org/abs/1905.02269 (2019).

[37] Abdelaal T, Mourragui S, Mahfouz A, Reinders MJT (2020). SpaGE: Spatial Gene Enhancement using scRNA-seq. Nucleic Acids Res..

[38] Cao K, Gong Q, Hong Y, Wan L (2022). A unified computational framework for single-cell data integration with optimal transport. Nat. Commun..

[39] Stickels RR (2021). Highly sensitive spatial transcriptomics at near-cellular resolution with Slide-seqV2. Nat. Biotechnol..

[40] Yang Y, Zhao H, Wang J, Zhou Y (2014). SPOT-Seq-RNA: predicting protein-RNA complex structure and RNA-binding function by fold recognition and binding affinity prediction. Methods Mol. Biol..

[41] Gouwens NW (2020). Integrated morphoelectric and transcriptomic classification of cortical GABAergic. Cells. Cell.

[42] Moses L, Pachter L (2022). Museum of spatial transcriptomics. Nat. Methods.

[43] Moor AE (2018). Spatial reconstruction of single enterocytes uncovers broad zonation along the intestinal villus axis. Cell.

[44] Lee BR (2021). Scaled, high fidelity electrophysiological, morphological, and transcriptomic cell characterization. eLife.

[45] Lipovsek M (2021). Patch-seq: Past, Present, and Future. J. Neurosci..

[46] Hwang B, Lee JH, Bang D (2018). Single-cell RNA sequencing technologies and bioinformatics pipelines. Exp. Mol. Med..

[47] Zhang MJ, Ntranos V, Tse D (2020). Determining sequencing depth in a single-cell RNA-seq experiment. Nat. Commun..

[48] Avila Cobos F, Alquicira-Hernandez J, Powell JE, Mestdagh P, De Preter K (2020). Benchmarking of cell type deconvolution pipelines for transcriptomics data. Nat. Commun..

[49] Fawkner-Corbett D (2021). Spatiotemporal analysis of human intestinal development at single-cell resolution. Cell.

[50] Ba, J. & Frey, B. Adaptive dropout for training deep neural networks. in *Advances in Neural Information Processing Systems 26* (eds. Burges, C. J. C., Bottou, L., Welling, M., Ghahramani, Z. & Weinberger, K. Q.) 3084–3092 (Curran Associates, Inc., 2013).

[51] Ioffe, S. & Szegedy, C. Batch Normalization: Accelerating Deep Network Training by Reducing Internal Covariate Shift. In *Proceedings of the 32nd International Conference on International Conference on Machine Learning*10.48550/arXiv.1502.03167**37**, 448–456 (2015).

[52] Kingma, D. P. & Ba, J. Adam: A Method for Stochastic Optimization. Preprint at 10.48550/arXiv.1412.6980 (2017).

[53] Burgess, C. P. et al. Understanding disentangling in $\beta$-VAE. Preprint at *arXiv:1804.03599 [cs, stat]* (2018).

[54] Berg J (2021). Human neocortical expansion involves glutamatergic neuron diversification. Nature.

[55] Darmanis S (2015). A survey of human brain transcriptome diversity at the single cell level. Proc. Natl Acad. Sci. USA.

[56] Singh, R. et al. Unsupervised manifold alignment for single-cell multi-omics data. in *Proceedings of the 11th ACM International Conference on Bioinformatics, Computational Biology and Health Informatics* 1–10 (ACM, 2020) 10.1145/3388440.3412410. 10.1145/3388440.3412410 PMC8095090 33954299

[57] Lopez R, Regier J, Cole MB, Jordan MI, Yosef N (2018). Deep generative modeling for single-cell transcriptomics. Nat. Methods.

[58] Johansen, N., Hu, H. & Quon, G. Projecting RNA measurements onto single cell atlases to extract cell type-specific expression profiles using scProjection. *scProjection*10.5281/zenodo.8173396 (2023). 10.1038/s41467-023-40744-6 PMC10457395 37626024

[59] Shannon P (2003). Cytoscape: a software environment for integrated models of biomolecular interaction networks. Genome Res..

