# Supplementary material for: Projecting RNA measurements onto single cell atlases to extract cell type-specific expression profiles using scProjection
Source: Nat Commun. 2023 Aug 25;14:5192. doi: 10.1038/s41467-023-40744-6 (PMC10457395; doi:10.1038/s41467-023-40744-6)
Supplement: Supplementary file 1 — Supplementary Information [file 41467_2023_40744_MOESM1_ESM.pdf]

Supplementary Information

Projecting RNA measurements onto single cell atlases to extract cell type-specific expression profiles

Nelson Johansen[1, *, †], Hongru Hu[2, †], Gerald Quon[1, 2, 3*]

[1]Graduate Group in Computer Science, University of California, Davis, Davis, CA, [2]Integrative Genetics and Genomics Graduate Group, University of California, Davis, Davis, CA, [3]Department of Molecular and Cellular Biology, University of California, Davis, Davis, CA

†These authors contributed equally to this work

*To whom correspondence should be addressed: njjohansen@ucdavis.edu, gquon@ucdavis.edu

Table of Contents

Fig S1. Benchmarking of spatial transcriptome deconvolution methods 2

Fig S2. Benchmarking of deconvolution methods on CellBench 3

Fig S3. Likelihood and proportion of CellBench mixtures, when scRNA-seq data for HCC827 is removed from the scProjection training atlas 4

Fig S4. scProjection accurately projects mixed RNA samples to cell type-specific expression profiles 5

Fig S5. Expression of cell type-specific marker genes in mouse Patch-seq data 6

Fig S6. Estimated cell type abundances of Patch-seq data using single cell atlas of the mouse cortex 7

Fig S7. Correlation of ion channel genes with electrophysiology features 8

Fig S8. Estimated cell type abundances and likelihood for ROSMAP bulk samples 9

Fig S9. Likelihood of CellBench mixtures under each component VAE in scProjection 10

Fig S10. Correlation of metrics used in benchmarking experiments 11

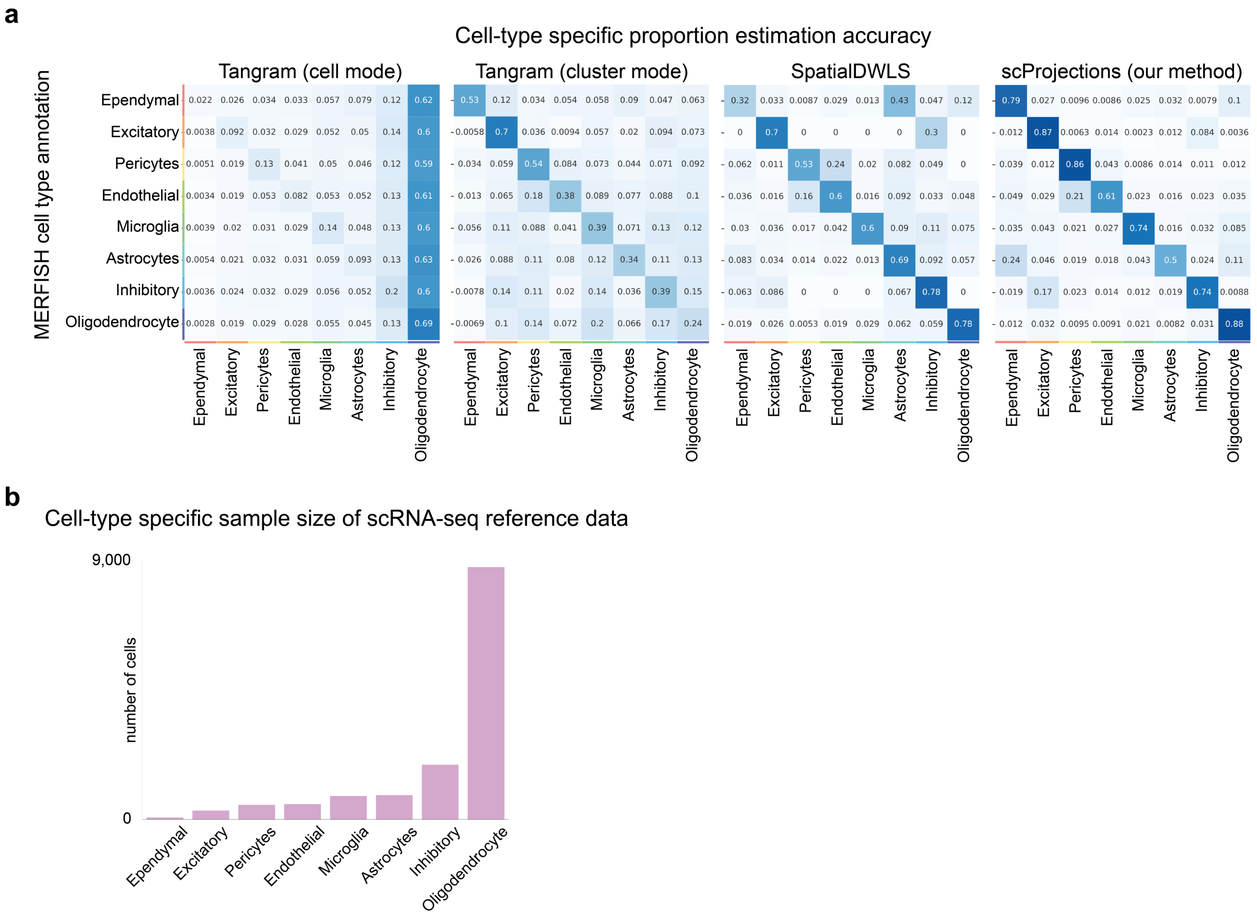

Fig S1. Benchmarking of spatial transcriptome deconvolution methods[1,2] **(a)** Heatmaps visualize the average predicted abundance of each cell type as defined in the single cell atlas (columns), for MERFISH RNA measurements that were categorized in the original study[3] based on the MERFISH measurements alone (rows). **(b)** Bar plot shows the number of cells of each cell type in the high-resolution reference scRNAseq data from the same MERFISH study.

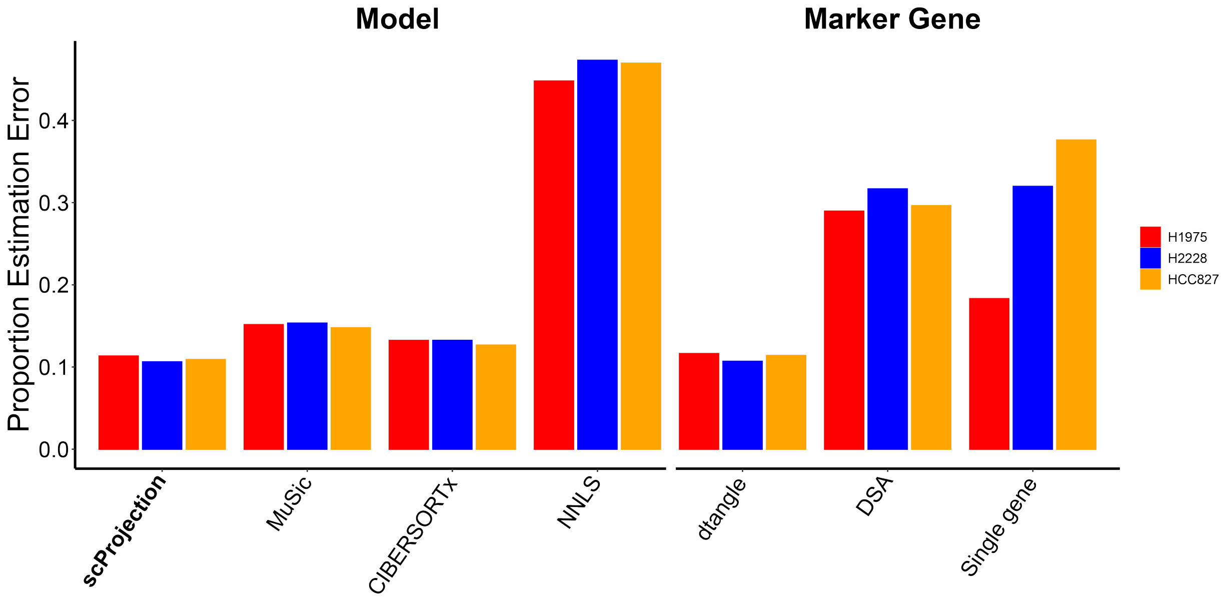

Fig S2. Benchmarking of deconvolution methods on CellBench[4] Bar plots indicate the error in predicted cell type abundances for each deconvolution method[5–8].

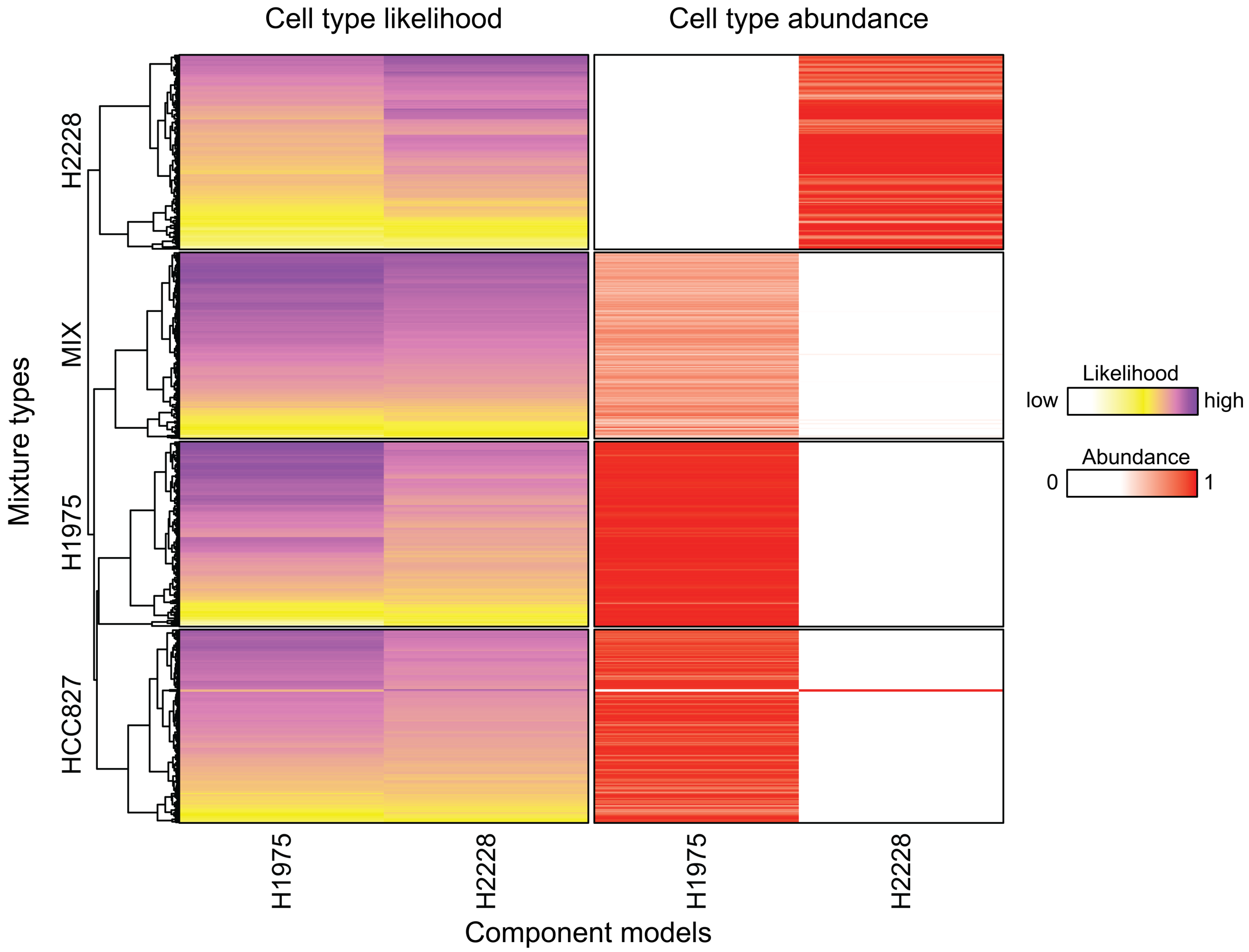

Fig S3. Likelihood and proportion of CellBench mixtures, when scRNA-seq data for HCC827 is removed from the scProjection training atlas Heatmaps visualizing the likelihood and estimated abundances for each mixture sample (row) under each component model (column), where mixture samples are again labeled by the cell type contributing the most RNA.

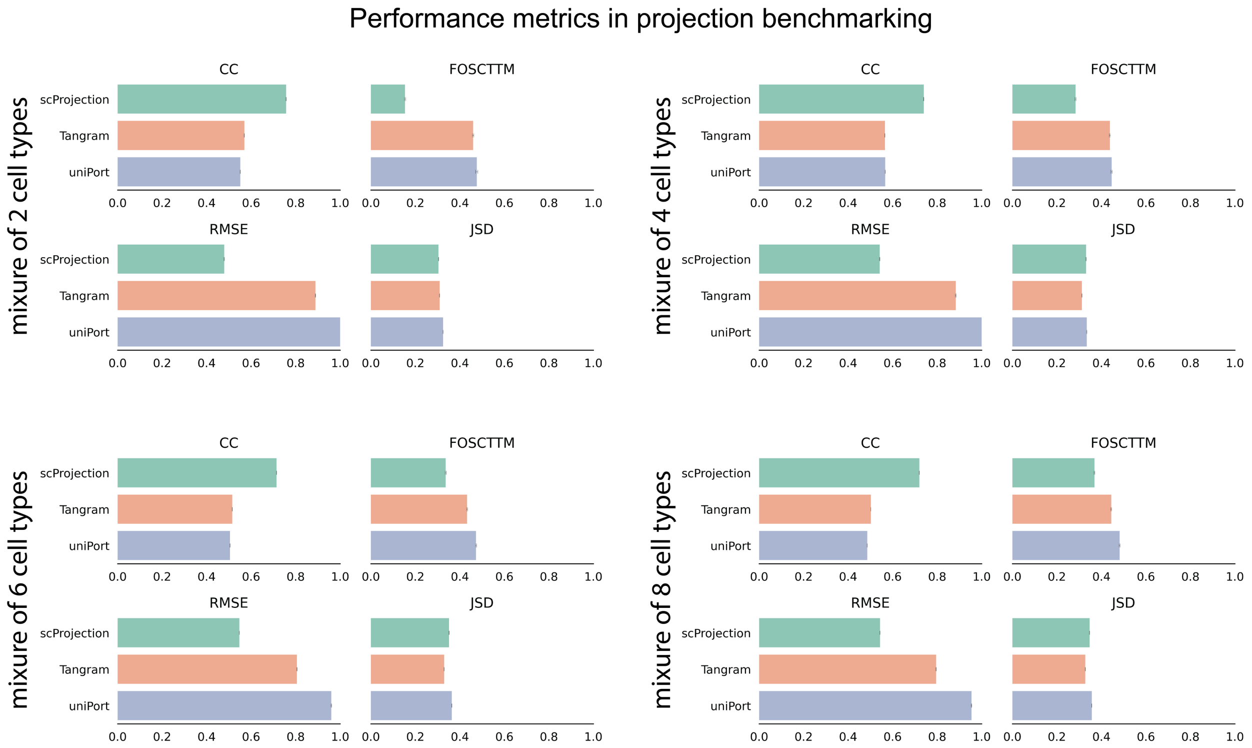

Fig S4. scProjection accurately projects mixed RNA samples to cell type-specific expression profiles Bar plots show the projection performance across different methods including scProjection, Tangram[1], and uniPort[9]. Performance is measured separately for each set of mixed RNA samples consisting of either 2, 4, 6 or 8 neuronal cell types mixed together. The benchmark metrics include average cell-wise correlation coefficients (CC), fraction of samples closer than the true match (FOSCTTM), Root Mean Square Error (RMSE), and Jensen-Shannon distances (JSD) between the projected RNA and ground truth single-cell RNA profiles[10,11] used to define the simulated RNA mixture.

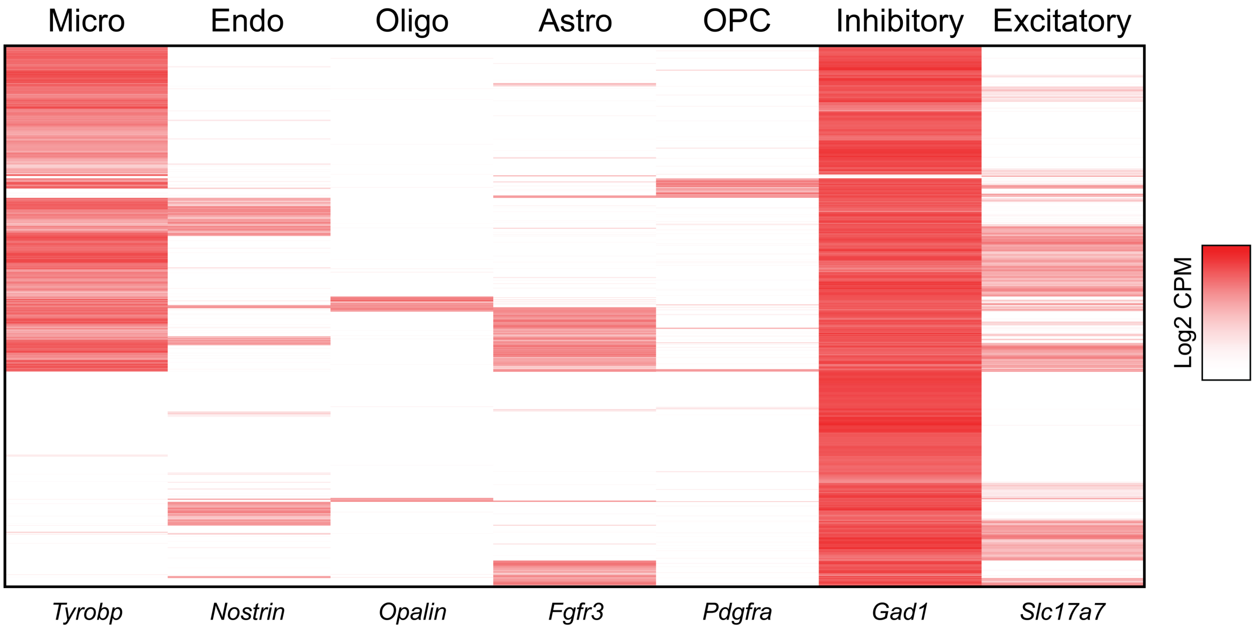

Fig S5. Expression of cell type-specific marker genes in mouse Patch-seq data[12]
Heatmaps visualize the log2 CPM expression of cell type-specific marker genes (columns) for each PatchSeq sample (rows).

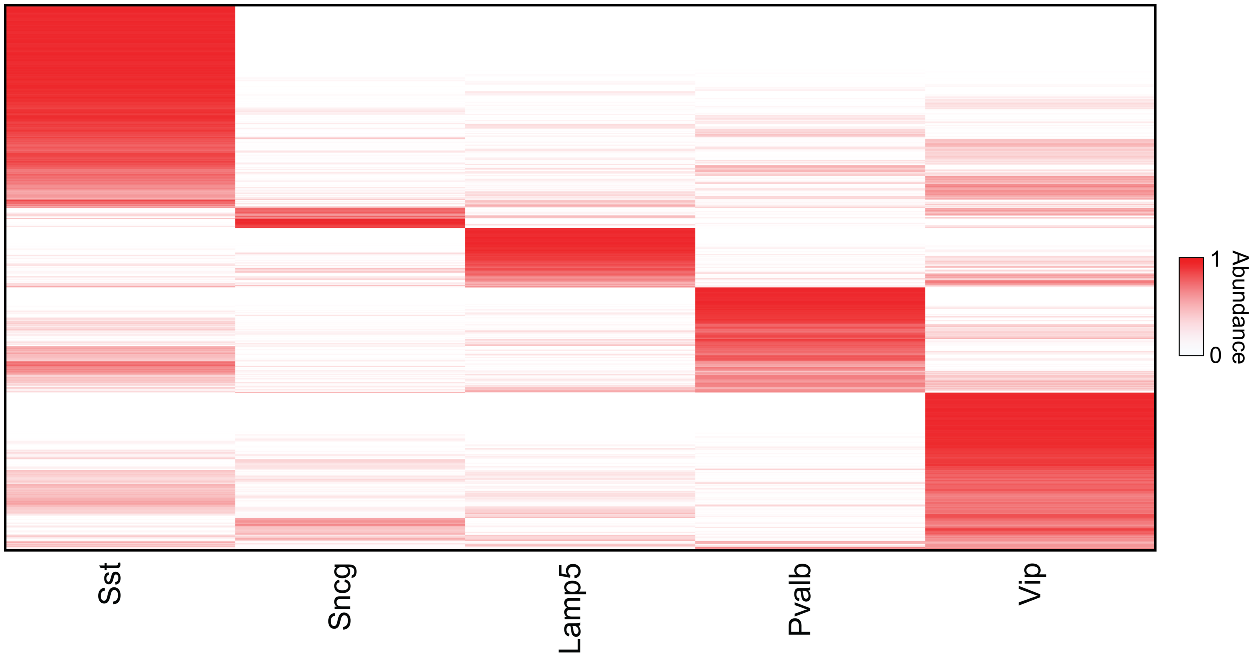

Fig S6. Estimated cell type abundances of Patch-seq data using single cell atlas of the mouse cortex[10] Heatmaps visualize the abundance of each GABAergic subclass (columns) for each PatchSeq sample (rows) based on training scProjection using the Yao et al. atlas.

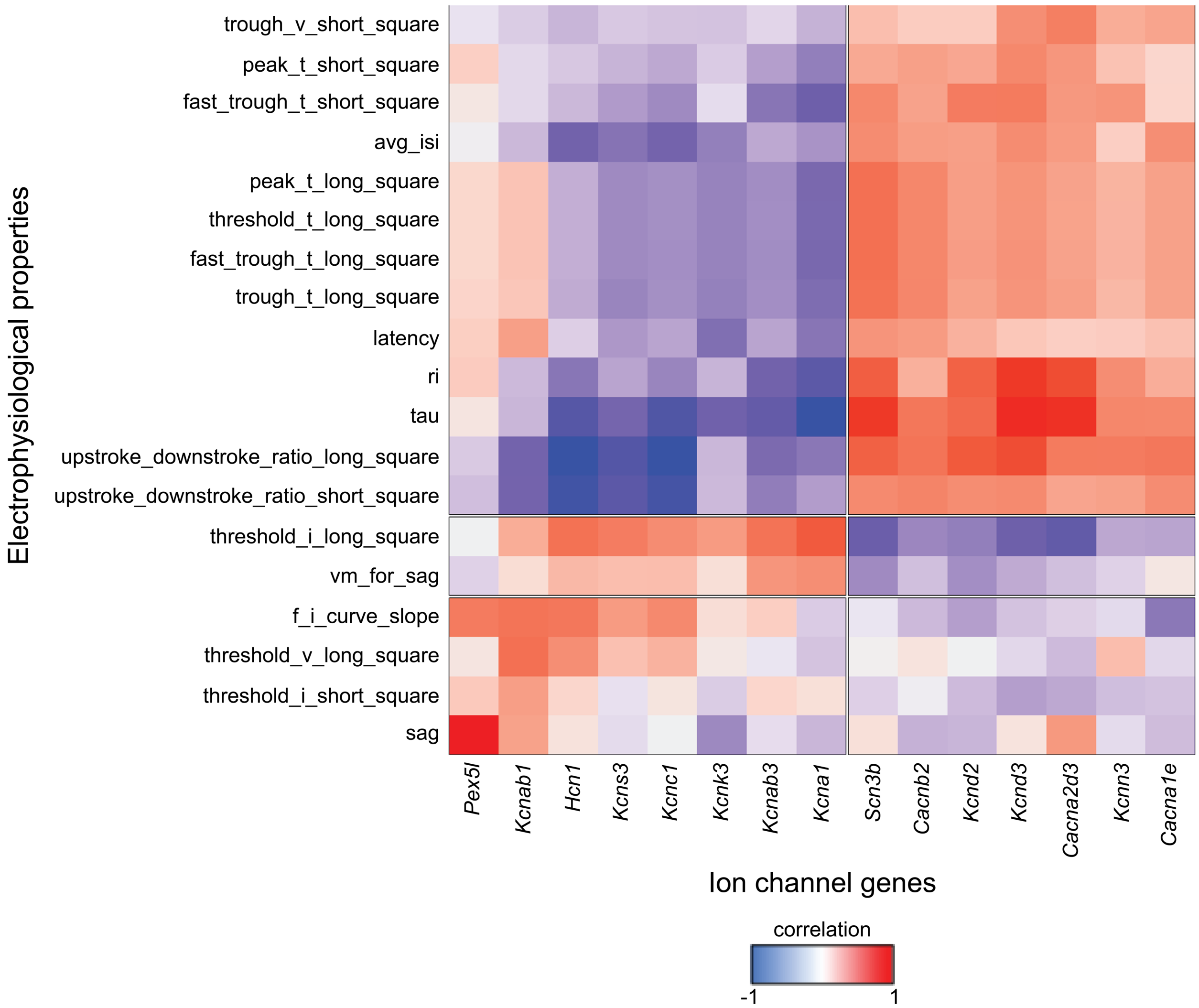

Fig S7. Correlation of ion channel genes with electrophysiology features Heatmap visualizes the correlation of the most variable ion channel genes that play a role in neuronal signaling (columns) with electrophysiology features (rows).

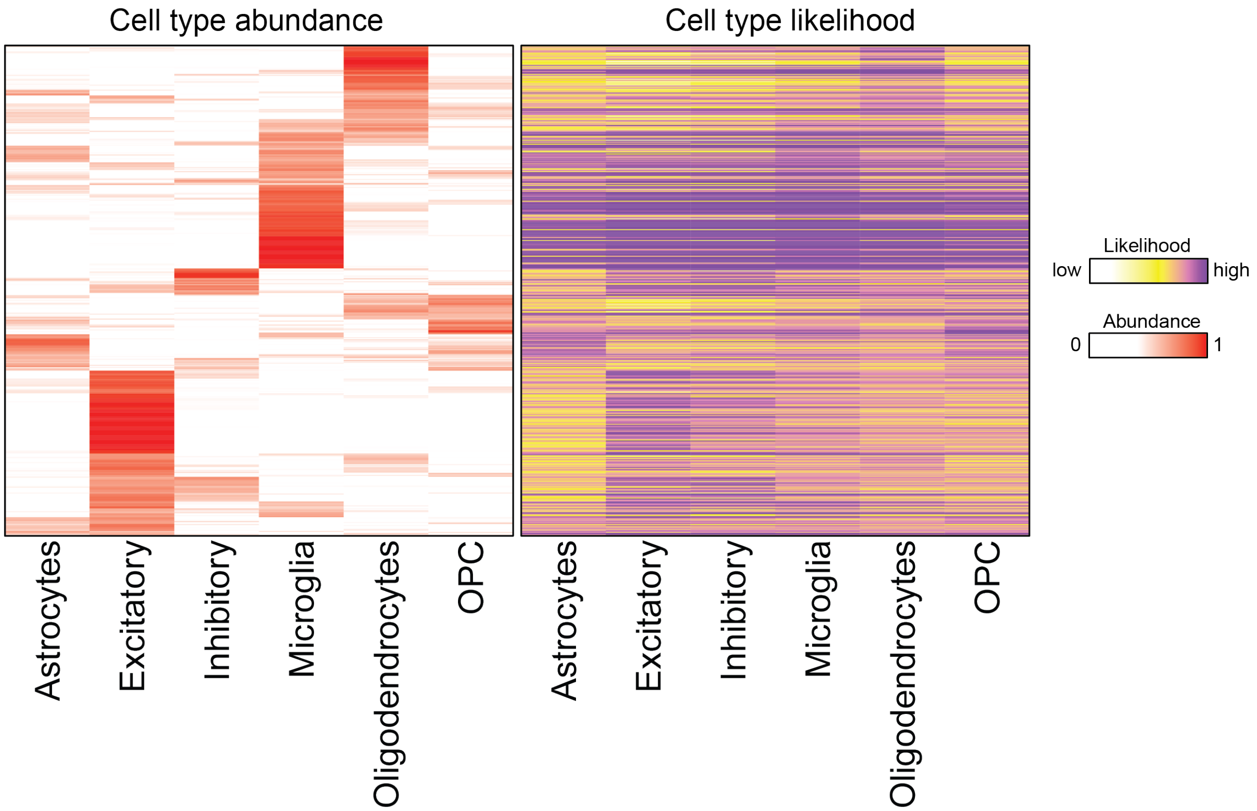

Fig S8. Estimated cell type abundances and likelihood for ROSMAP bulk samples[13] The left heatmap indicates the estimated abundances of each cell type (columns) for each bulk sample (rows), and the right heatmap shows the likelihood of each mixed sample under each VAE for a given cell type (columns).

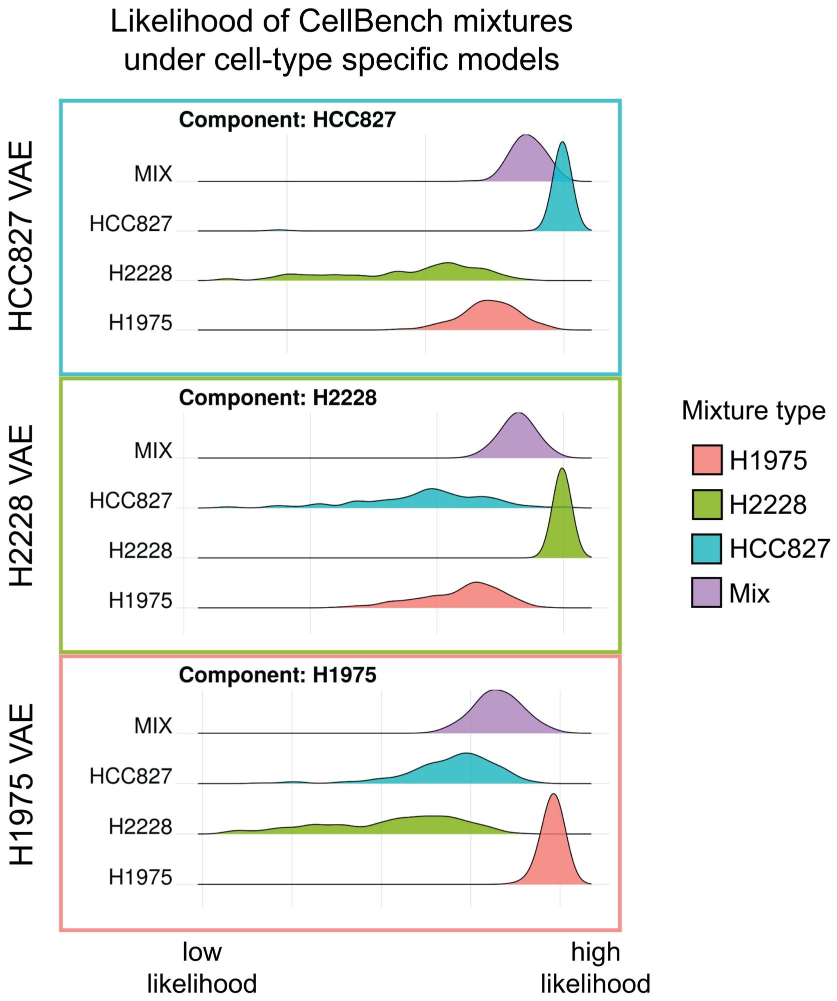

Fig S9. Likelihood of CellBench mixtures under each component VAE in scProjection
Density plots for each component VAE (bounded boxes) indicate the likelihood of each mixture type under the corresponding cell type model trained by scProjection.

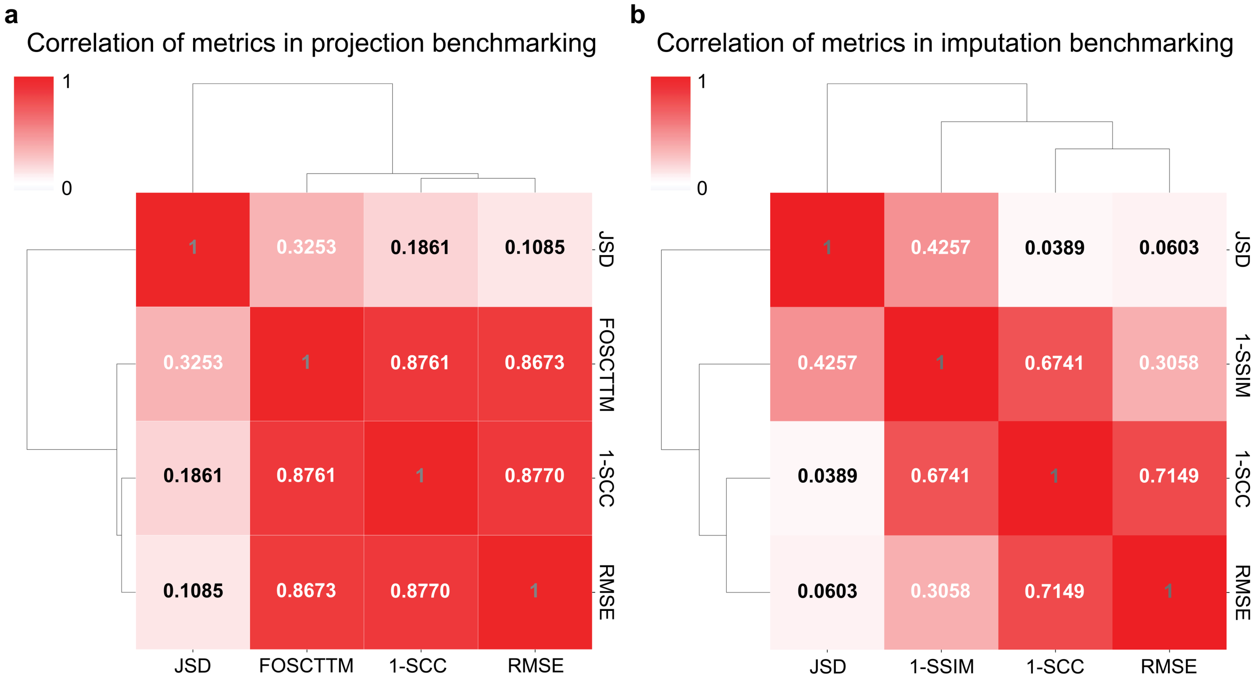

Fig S10. Correlation of metrics used in benchmarking experiments (a) Heatmap indicates the positive correlation between JSD, FOSCTTM, and RMSE (the smaller the better), and negative correlation between the three metrics and SCC (the larger the better) in the projection benchmarking experiment (**Fig. 2**). **(b)** Heatmap indicates the positive correlation between JSD and RMSE (the smaller the better), and between SSIM and SCC (the larger the better) respectively in the imputation experiment (**Fig. 3**).

Supplementary References

1. Biancalani, T. *et al.* Deep learning and alignment of spatially resolved single-cell transcriptomes with Tangram. *Nat Methods* **18**, 1352–1362 (2021).
2. Dong, R. & Yuan, G.-C. SpatialDWLS: accurate deconvolution of spatial transcriptomic data. *Genome Biol* **22**, 145 (2021).
3. Moffitt, J. R. *et al.* Molecular, spatial, and functional single-cell profiling of the hypothalamic preoptic region. *Science* **362**, (2018).
4. Tian, L. *et al.* Benchmarking single cell RNA-sequencing analysis pipelines using mixture control experiments. *Nat Methods* **16**, 479–487 (2019).
5. Newman, A. M. *et al.* Determining cell type abundance and expression from bulk tissues with digital cytometry. *Nat Biotechnol* **37**, 773–782 (2019).
6. Wang, X., Park, J., Susztak, K., Zhang, N. R. & Li, M. Bulk tissue cell type deconvolution with multi-subject single-cell expression reference. *Nat Commun* **10**, 380 (2019).
7. Hunt, G. J., Freytag, S., Bahlo, M. & Gagnon-Bartsch, J. A. dtangle: accurate and robust cell type deconvolution. *Bioinformatics* **35**, 2093–2099 (2019).
8. Zhong, Y., Wan, Y.-W., Pang, K., Chow, L. M. & Liu, Z. Digital sorting of complex tissues for cell type-specific gene expression profiles. *BMC Bioinformatics* **14**, 89 (2013).
9. Cao, K., Gong, Q., Hong, Y. & Wan, L. A unified computational framework for single-cell data integration with optimal transport. *Nat Commun* **13**, 7419 (2022).
10. Yao, Z. *et al.* A transcriptomic and epigenomic cell atlas of the mouse primary motor cortex. *Nature* **598**, 103–110 (2021).
11. Yao, Z. *et al.* A taxonomy of transcriptomic cell types across the isocortex and hippocampal formation. *Cell* **184**, 3222-3241.e26 (2021).
12. Gouwens, N. W. *et al.* Integrated Morphoelectric and Transcriptomic Classification of Cortical GABAergic Cells. *Cell* **183**, 935-953.e19 (2020).
13. Bennett, D. A. *et al.* Religious Orders Study and Rush Memory and Aging Project. *JAD* **64**, S161–S189 (2018).
