# Supplementary material for: Projecting RNA measurements onto single cell atlases to extract cell type-specific expression profiles using scProjection
Source: Nat Commun. 2023 Aug 25;14:5192. doi: 10.1038/s41467-023-40744-6 (PMC10457395; doi:10.1038/s41467-023-40744-6)
Supplement: Supplementary file 2 — Reporting Summary [file 41467_2023_40744_MOESM2_ESM.pdf]

Corresponding author(s): *Double-anonymous peer review submissions: write DAPR and your manuscript number here instead of author names.*

Last updated by author(s): *YYYY-MM-DD*

Reporting Summary

Nature Portfolio wishes to improve the reproducibility of the work that we publish. This form provides structure for consistency and transparency in reporting. For further information on Nature Portfolio policies, see our Editorial Policies and the Editorial Policy Checklist.

Statistics

For all statistical analyses, confirm that the following items are present in the figure legend, table legend, main text, or Methods section.

n/a	Confirmed	
☐	☒	The exact sample size (*n*) for each experimental group/condition, given as a discrete number and unit of measurement
☐	☒	A statement on whether measurements were taken from distinct samples or whether the same sample was measured repeatedly
☒	☐	The statistical test(s) used AND whether they are one- or two-sided<br>*Only common tests should be described solely by name; describe more complex techniques in the Methods section.*
☐	☒	A description of all covariates tested
☐	☒	A description of any assumptions or corrections, such as tests of normality and adjustment for multiple comparisons
☐	☒	A full description of the statistical parameters including central tendency (e.g. means) or other basic estimates (e.g. regression coefficient) AND variation (e.g. standard deviation) or associated estimates of uncertainty (e.g. confidence intervals)
☒	☐	For null hypothesis testing, the test statistic (e.g. *F*, *t*, *r*) with confidence intervals, effect sizes, degrees of freedom and *P* value noted<br>*Give P values as exact values whenever suitable.*
☒	☐	For Bayesian analysis, information on the choice of priors and Markov chain Monte Carlo settings
☒	☐	For hierarchical and complex designs, identification of the appropriate level for tests and full reporting of outcomes
☐	☒	Estimates of effect sizes (e.g. Cohen's *d*, Pearson's *r*), indicating how they were calculated

Our web collection on statistics for biologists contains articles on many of the points above.

Software and code

Policy information about availability of computer code

Data collection	No software was used for data collection.
Data analysis	The scProjection framework was implemented in the ‘scProjection’ Python package, which can be installed through PyPI (https://pypi.org/project/scProjection/), and the code is available at https://github.com/quon-titative-biology/scProjection. The data preprocessing and analysis of results were done using R 3.6.1 and R 4.0.1, Seurat v4.3.0 and Python 3.8.13. Other software used in this paper: scanpy (v1.9.3), gimVI from scvi (v0.20.3), uniport (v1.1.2), tangram (v1.0.4), SpatialDWLS (from Giotto v1.1.0), MuSiC (v0.1.1), DSA (v0.74.18), and dtangle (v2.0.9). CIBERSORTx was run from their website (https://cibersortx.stanford.edu/), which did not provide versioning information at runtime.

For manuscripts utilizing custom algorithms or software that are central to the research but not yet described in published literature, software must be made available to editors and reviewers. We strongly encourage code deposition in a community repository (e.g. GitHub). See the Nature Portfolio guidelines for submitting code & software for further information.

Data

Policy information about availability of data

All manuscripts must include a data availability statement. This statement should provide the following information, where applicable:

- Accession codes, unique identifiers, or web links for publicly available datasets
- A description of any restrictions on data availability
- For clinical datasets or third party data, please ensure that the statement adheres to our policy

All data analyzed in this article are publicly available through online sources. The gene count matrix for the RNA mixture experiments in CellBench is provided in the

R data file that is available at https://github.com/Shians/CellBench. The gene count matrix of the bulk-RNA experiments and IHC measurements for the ROSMAP-IHC benchmark can be found at https://github.com/ellispatrick/CortexCellDeconv. Mouse Primary Motor Area (MOp) and the mouse primary visual cortex (VISp) scRNA-seq datasets are from the Cell Types Database of the Allen Brain Map (https://portal.brain-map.org/atlases-and-data/rnaseq/mouse-aca-and-mop-smart-seq, and https://portal.brain-map.org/atlases-and-data/rnaseq/mouse-v1-and-alm-smart-seq, respectively). We obtained the gene count matrix for the mouse brain atlas described in Yao et al. and Tasic et al. from the Allen Institute Cell Types database: RNA-Seq data page on the Allen Institute's webpage (https://portal.brain-map.org/atlases-and-data/rnaseq). The MERFISH gene luminescence matrix described in Moffitt et al. can be accessed from DRYAD (https://doi.org/10.5061/dryad.8t8s248) and the scRNA-seq count matrix can be found at NCBI Gene Expression Omnibus (GEO) database with accession ID: GSE113576 (https://www.ncbi.nlm.nih.gov/geo/query/acc.cgi?acc=GSE113576). The gene expression matrices for the LCM-seq, scRNA-seq and spatial reconstructions experiments described in Moor et al. can be found at NCBI GEO database with accession ID: GSE109413 (https://www.ncbi.nlm.nih.gov/geo/query/acc.cgi?acc=GSE109413) and Zenodo open repository https://doi.org/10.5281/zenodo.1320734. The gene count matrices for mouse and human cortex Patch-seq experiments are available at https://portal.brain-map.org/explore/classes/multimodal-characterization, and the paired electrophysiological recording datasets are available at The DANDI Archive site with ID: 000020 (https://dandiarchive.org/dandiset/000020) and ID: 000023 (https://dandiarchive.org/dandiset/000023), respectively. The gene count matrices of Patch-seq studies from the Foldy et al. and Cadwell et al. have been deposited at NCBI GEO database with accession ID: GSE75386 (https://www.ncbi.nlm.nih.gov/geo/query/acc.cgi?acc=GSE75386), and https://www.ebi.ac.uk/biostudies/arrayexpress/studies/E-MTAB-4092 respectively. Source data are provided as a Source Data file.

Research involving human participants, their data, or biological material

Policy information about studies with human participants or human data. See also policy information about sex, gender (identity/presentation), and sexual orientation and race, ethnicity and racism.

Reporting on sex and gender	N/A
Reporting on race, ethnicity, or other socially relevant groupings	N/A
Population characteristics	N/A
Recruitment	N/A
Ethics oversight	N/A

Note that full information on the approval of the study protocol must also be provided in the manuscript.

Field-specific reporting

Please select the one below that is the best fit for your research. If you are not sure, read the appropriate sections before making your selection.

☒ Life sciences ☐ Behavioural & social sciences ☐ Ecological, evolutionary & environmental sciences

For a reference copy of the document with all sections, see nature.com/documents/nr-reporting-summary-flat.pdf

Life sciences study design

All studies must disclose on these points even when the disclosure is negative.

Sample size	We applied and benchmarked scProjection using seven publicly available, bulk- (or bulk-like) expression datasets from diverse assays, including bulk RNA sequencing, RNA imaging-based MERFISH, LCM-seq, and Patch-seq. Our results are therefore based on reasonable sample sizes.
Data exclusions	No additional sample filtering steps were applied to the datasets obtained from the public domain.
Replication	As the entire paper is based on computational analysis, reproducibility of results was ensured by repeating our analysis scripts to ensure the same results were reproduced for each figure.
Randomization	Randomization was not performed in this study. In each experiment, scProjection is trained on an entire dataset of bulk- (or bulk-like) RNA samples, and is only given a sample single cell reference dataset to help train the VAEs. Because the bulk RNA samples are not labeled in any way when input into scProjection, there was no need to define a training/testing split of the dataset.
Blinding	Blinding is not relevant to this study, as there was no explicit randomization.

Reporting for specific materials, systems and methods

We require information from authors about some types of materials, experimental systems and methods used in many studies. Here, indicate whether each material, system or method listed is relevant to your study. If you are not sure if a list item applies to your research, read the appropriate section before selecting a response.

Materials & experimental systems

n/a	Involved in the study
☒	☐ Antibodies
☒	☐ Eukaryotic cell lines
☒	☐ Palaeontology and archaeology
☒	☐ Animals and other organisms
☒	☐ Clinical data
☒	☐ Dual use research of concern
☒	☐ Plants

Methods

n/a	Involved in the study
☒	☐ ChIP-seq
☒	☐ Flow cytometry
☒	☐ MRI-based neuroimaging
